# Search for direct top squark pair production in events with a $Z$ boson, $b$-jets and missing transverse momentum in $\sqrt{s}=8$ TeV $pp$ collisions with the ATLAS detector

**DOI:** 10.1140/epjc/s10052-014-2883-6

**Published:** 2014-06-03

**Authors:** The ATLAS Collaboration, G. Aad, B. Abbott, J. Abdallah, S. Abdel Khalek, O. Abdinov, R. Aben, B. Abi, M. Abolins, O. S. AbouZeid, H. Abramowicz, H. Abreu, R. Abreu, Y. Abulaiti, B. S. Acharya, L. Adamczyk, D. L. Adams, J. Adelman, S. Adomeit, T. Adye, T. Agatonovic-Jovin, J. A. Aguilar-Saavedra, M. Agustoni, S. P. Ahlen, A. Ahmad, F. Ahmadov, G. Aielli, T. P. A. Åkesson, G. Akimoto, A. V. Akimov, G. L. Alberghi, J. Albert, S. Albrand, M. J. Alconada Verzini, M. Aleksa, I. N. Aleksandrov, C. Alexa, G. Alexander, G. Alexandre, T. Alexopoulos, M. Alhroob, G. Alimonti, L. Alio, J. Alison, B. M. M. Allbrooke, L. J. Allison, P. P. Allport, S. E. Allwood-Spiers, J. Almond, A. Aloisio, A. Alonso, F. Alonso, C. Alpigiani, A. Altheimer, B. Alvarez Gonzalez, M. G. Alviggi, K. Amako, Y. Amaral Coutinho, C. Amelung, D. Amidei, S. P. Amor Dos Santos, A. Amorim, S. Amoroso, N. Amram, G. Amundsen, C. Anastopoulos, L. S. Ancu, N. Andari, T. Andeen, C. F. Anders, G. Anders, K. J. Anderson, A. Andreazza, V. Andrei, X. S. Anduaga, S. Angelidakis, I. Angelozzi, P. Anger, A. Angerami, F. Anghinolfi, A. V. Anisenkov, N. Anjos, A. Annovi, A. Antonaki, M. Antonelli, A. Antonov, J. Antos, F. Anulli, M. Aoki, L. Aperio Bella, R. Apolle, G. Arabidze, I. Aracena, Y. Arai, J. P. Araque, A. T. H. Arce, J.-F. Arguin, S. Argyropoulos, M. Arik, A. J. Armbruster, O. Arnaez, V. Arnal, H. Arnold, O. Arslan, A. Artamonov, G. Artoni, S. Asai, N. Asbah, A. Ashkenazi, S. Ask, B. Åsman, L. Asquith, K. Assamagan, R. Astalos, M. Atkinson, N. B. Atlay, B. Auerbach, K. Augsten, M. Aurousseau, G. Avolio, G. Azuelos, Y. Azuma, M. A. Baak, C. Bacci, H. Bachacou, K. Bachas, M. Backes, M. Backhaus, J. Backus Mayes, E. Badescu, P. Bagiacchi, P. Bagnaia, Y. Bai, T. Bain, J. T. Baines, O. K. Baker, S. Baker, P. Balek, F. Balli, E. Banas, Sw. Banerjee, D. Banfi, A. Bangert, A. A. E. Bannoura, V. Bansal, H. S. Bansil, L. Barak, S. P. Baranov, E. L. Barberio, D. Barberis, M. Barbero, T. Barillari, M. Barisonzi, T. Barklow, N. Barlow, B. M. Barnett, R. M. Barnett, Z. Barnovska, A. Baroncelli, G. Barone, A. J. Barr, F. Barreiro, J. Barreiro Guimarães da Costa, R. Bartoldus, A. E. Barton, P. Bartos, V. Bartsch, A. Bassalat, A. Basye, R. L. Bates, L. Batkova, J. R. Batley, M. Battistin, F. Bauer, H. S. Bawa, T. Beau, P. H. Beauchemin, R. Beccherle, P. Bechtle, H. P. Beck, K. Becker, S. Becker, M. Beckingham, C. Becot, A. J. Beddall, A. Beddall, S. Bedikian, V. A. Bednyakov, C. P. Bee, L. J. Beemster, T. A. Beermann, M. Begel, K. Behr, C. Belanger-Champagne, P. J. Bell, W. H. Bell, G. Bella, L. Bellagamba, A. Bellerive, M. Bellomo, A. Belloni, O. L. Beloborodova, K. Belotskiy, O. Beltramello, O. Benary, D. Benchekroun, K. Bendtz, N. Benekos, Y. Benhammou, E. Benhar Noccioli, J. A. Benitez Garcia, D. P. Benjamin, J. R. Bensinger, K. Benslama, S. Bentvelsen, D. Berge, E. Bergeaas Kuutmann, N. Berger, F. Berghaus, E. Berglund, J. Beringer, C. Bernard, P. Bernat, C. Bernius, F. U. Bernlochner, T. Berry, P. Berta, C. Bertella, F. Bertolucci, M. I. Besana, G. J. Besjes, O. Bessidskaia, N. Besson, C. Betancourt, S. Bethke, W. Bhimji, R. M. Bianchi, L. Bianchini, M. Bianco, O. Biebel, S. P. Bieniek, K. Bierwagen, J. Biesiada, M. Biglietti, J. Bilbao De Mendizabal, H. Bilokon, M. Bindi, S. Binet, A. Bingul, C. Bini, C. W. Black, J. E. Black, K. M. Black, D. Blackburn, R. E. Blair, J.-B. Blanchard, T. Blazek, I. Bloch, C. Blocker, W. Blum, U. Blumenschein, G. J. Bobbink, V. S. Bobrovnikov, S. S. Bocchetta, A. Bocci, C. R. Boddy, M. Boehler, J. Boek, T. T. Boek, J. A. Bogaerts, A. G. Bogdanchikov, A. Bogouch, C. Bohm, J. Bohm, V. Boisvert, T. Bold, V. Boldea, A. S. Boldyrev, M. Bomben, M. Bona, M. Boonekamp, A. Borisov, G. Borissov, M. Borri, S. Borroni, J. Bortfeldt, V. Bortolotto, K. Bos, D. Boscherini, M. Bosman, H. Boterenbrood, J. Boudreau, J. Bouffard, E. V. Bouhova-Thacker, D. Boumediene, C. Bourdarios, N. Bousson, S. Boutouil, A. Boveia, J. Boyd, I. R. Boyko, I. Bozovic-Jelisavcic, J. Bracinik, P. Branchini, A. Brandt, G. Brandt, O. Brandt, U. Bratzler, B. Brau, J. E. Brau, H. M. Braun, S. F. Brazzale, B. Brelier, K. Brendlinger, A. J. Brennan, R. Brenner, S. Bressler, K. Bristow, T. M. Bristow, D. Britton, F. M. Brochu, I. Brock, R. Brock, C. Bromberg, J. Bronner, G. Brooijmans, T. Brooks, W. K. Brooks, J. Brosamer, E. Brost, G. Brown, J. Brown, P. A. Bruckman de Renstrom, D. Bruncko, R. Bruneliere, S. Brunet, A. Bruni, G. Bruni, M. Bruschi, L. Bryngemark, T. Buanes, Q. Buat, F. Bucci, P. Buchholz, R. M. Buckingham, A. G. Buckley, S. I. Buda, I. A. Budagov, F. Buehrer, L. Bugge, M. K. Bugge, O. Bulekov, A. C. Bundock, H. Burckhart, S. Burdin, B. Burghgrave, S. Burke, I. Burmeister, E. Busato, D. Büscher, V. Büscher, P. Bussey, C. P. Buszello, B. Butler, J. M. Butler, A. I. Butt, C. M. Buttar, J. M. Butterworth, P. Butti, W. Buttinger, A. Buzatu, M. Byszewski, S. Cabrera Urbán, D. Caforio, O. Cakir, P. Calafiura, A. Calandri, G. Calderini, P. Calfayan, R. Calkins, L. P. Caloba, D. Calvet, S. Calvet, R. Camacho Toro, S. Camarda, D. Cameron, L. M. Caminada, R. Caminal Armadans, S. Campana, M. Campanelli, A. Campoverde, V. Canale, A. Canepa, J. Cantero, R. Cantrill, T. Cao, M. D. M. Capeans Garrido, I. Caprini, M. Caprini, M. Capua, R. Caputo, R. Cardarelli, T. Carli, G. Carlino, L. Carminati, S. Caron, E. Carquin, G. D. Carrillo-Montoya, A. A. Carter, J. R. Carter, J. Carvalho, D. Casadei, M. P. Casado, E. Castaneda-Miranda, A. Castelli, V. Castillo Gimenez, N. F. Castro, P. Catastini, A. Catinaccio, J. R. Catmore, A. Cattai, G. Cattani, S. Caughron, V. Cavaliere, D. Cavalli, M. Cavalli-Sforza, V. Cavasinni, F. Ceradini, B. Cerio, K. Cerny, A. S. Cerqueira, A. Cerri, L. Cerrito, F. Cerutti, M. Cerv, A. Cervelli, S. A. Cetin, A. Chafaq, D. Chakraborty, I. Chalupkova, K. Chan, P. Chang, B. Chapleau, J. D. Chapman, D. Charfeddine, D. G. Charlton, C. C. Chau, C. A. Chavez Barajas, S. Cheatham, A. Chegwidden, S. Chekanov, S. V. Chekulaev, G. A. Chelkov, M. A. Chelstowska, C. Chen, H. Chen, K. Chen, L. Chen, S. Chen, X. Chen, Y. Chen, H. C. Cheng, Y. Cheng, A. Cheplakov, R. Cherkaoui El Moursli, V. Chernyatin, E. Cheu, L. Chevalier, V. Chiarella, G. Chiefari, J. T. Childers, A. Chilingarov, G. Chiodini, A. S. Chisholm, R. T. Chislett, A. Chitan, M. V. Chizhov, S. Chouridou, B. K. B. Chow, I. A. Christidi, D. Chromek-Burckhart, M. L. Chu, J. Chudoba, J. J. Chwastowski, L. Chytka, G. Ciapetti, A. K. Ciftci, R. Ciftci, D. Cinca, V. Cindro, A. Ciocio, P. Cirkovic, Z. H. Citron, M. Citterio, M. Ciubancan, A. Clark, P. J. Clark, R. N. Clarke, W. Cleland, J. C. Clemens, C. Clement, Y. Coadou, M. Cobal, A. Coccaro, J. Cochran, L. Coffey, J. G. Cogan, J. Coggeshall, B. Cole, S. Cole, A. P. Colijn, C. Collins-Tooth, J. Collot, T. Colombo, G. Colon, G. Compostella, P. Conde Muiño, E. Coniavitis, M. C. Conidi, S. H. Connell, I. A. Connelly, S. M. Consonni, V. Consorti, S. Constantinescu, C. Conta, G. Conti, F. Conventi, M. Cooke, B. D. Cooper, A. M. Cooper-Sarkar, N. J. Cooper-Smith, K. Copic, T. Cornelissen, M. Corradi, F. Corriveau, A. Corso-Radu, A. Cortes-Gonzalez, G. Cortiana, G. Costa, M. J. Costa, D. Costanzo, D. Côté, G. Cottin, G. Cowan, B. E. Cox, K. Cranmer, G. Cree, S. Crépé-Renaudin, F. Crescioli, M. Crispin Ortuzar, M. Cristinziani, V. Croft, G. Crosetti, C.-M. Cuciuc, C. Cuenca Almenar, T. Cuhadar Donszelmann, J. Cummings, M. Curatolo, C. Cuthbert, H. Czirr, P. Czodrowski, Z. Czyczula, S. D’Auria, M. D’Onofrio, M. J. Da Cunha Sargedas De Sousa, C. Da Via, W. Dabrowski, A. Dafinca, T. Dai, O. Dale, F. Dallaire, C. Dallapiccola, M. Dam, A. C. Daniells, M. Dano Hoffmann, V. Dao, G. Darbo, G. L. Darlea, S. Darmora, J. A. Dassoulas, A. Dattagupta, W. Davey, C. David, T. Davidek, E. Davies, M. Davies, O. Davignon, A. R. Davison, P. Davison, Y. Davygora, E. Dawe, I. Dawson, R. K. Daya-Ishmukhametova, K. De, R. de Asmundis, S. De Castro, S. De Cecco, J. de Graat, N. De Groot, P. de Jong, H. De la Torre, F. De Lorenzi, L. De Nooij, D. De Pedis, A. De Salvo, U. De Sanctis, A. De Santo, J. B. De Vivie De Regie, G. De Zorzi, W. J. Dearnaley, R. Debbe, C. Debenedetti, B. Dechenaux, D. V. Dedovich, J. Degenhardt, I. Deigaard, J. Del Peso, T. Del Prete, F. Deliot, C. M. Delitzsch, M. Deliyergiyev, A. Dell’Acqua, L. Dell’Asta, M. Dell’Orso, M. Della Pietra, D. della Volpe, M. Delmastro, P. A. Delsart, C. Deluca, S. Demers, M. Demichev, A. Demilly, S. P. Denisov, D. Derendarz, J. E. Derkaoui, F. Derue, P. Dervan, K. Desch, C. Deterre, P. O. Deviveiros, A. Dewhurst, S. Dhaliwal, A. Di Ciaccio, L. Di Ciaccio, A. Di Domenico, C. Di Donato, A. Di Girolamo, B. Di Girolamo, A. Di Mattia, B. Di Micco, R. Di Nardo, A. Di Simone, R. Di Sipio, D. Di Valentino, M. A. Diaz, E. B. Diehl, J. Dietrich, T. A. Dietzsch, S. Diglio, A. Dimitrievska, J. Dingfelder, C. Dionisi, P. Dita, S. Dita, F. Dittus, F. Djama, T. Djobava, M. A. B. do Vale, A. Do Valle Wemans, T. K. O. Doan, D. Dobos, E. Dobson, C. Doglioni, T. Doherty, T. Dohmae, J. Dolejsi, Z. Dolezal, B. A. Dolgoshein, M. Donadelli, S. Donati, P. Dondero, J. Donini, J. Dopke, A. Doria, A. Dos Anjos, M. T. Dova, A. T. Doyle, M. Dris, J. Dubbert, S. Dube, E. Dubreuil, E. Duchovni, G. Duckeck, O. A. Ducu, D. Duda, A. Dudarev, F. Dudziak, L. Duflot, L. Duguid, M. Dührssen, M. Dunford, H. Duran Yildiz, M. Düren, A. Durglishvili, M. Dwuznik, M. Dyndal, J. Ebke, W. Edson, N. C. Edwards, W. Ehrenfeld, T. Eifert, G. Eigen, K. Einsweiler, T. Ekelof, M. El Kacimi, M. Ellert, S. Elles, F. Ellinghaus, N. Ellis, J. Elmsheuser, M. Elsing, D. Emeliyanov, Y. Enari, O. C. Endner, M. Endo, R. Engelmann, J. Erdmann, A. Ereditato, D. Eriksson, G. Ernis, J. Ernst, M. Ernst, J. Ernwein, D. Errede, S. Errede, E. Ertel, M. Escalier, H. Esch, C. Escobar, B. Esposito, A. I. Etienvre, E. Etzion, H. Evans, L. Fabbri, G. Facini, R. M. Fakhrutdinov, S. Falciano, Y. Fang, M. Fanti, A. Farbin, A. Farilla, T. Farooque, S. Farrell, S. M. Farrington, P. Farthouat, F. Fassi, P. Fassnacht, D. Fassouliotis, A. Favareto, L. Fayard, P. Federic, O. L. Fedin, W. Fedorko, M. Fehling-Kaschek, S. Feigl, L. Feligioni, C. Feng, E. J. Feng, H. Feng, A. B. Fenyuk, S. Fernandez Perez, S. Ferrag, J. Ferrando, A. Ferrari, P. Ferrari, R. Ferrari, D. E. Ferreira de Lima, A. Ferrer, D. Ferrere, C. Ferretti, A. Ferretto Parodi, M. Fiascaris, F. Fiedler, A. Filipčič, M. Filipuzzi, F. Filthaut, M. Fincke-Keeler, K. D. Finelli, M. C. N. Fiolhais, L. Fiorini, A. Firan, J. Fischer, W. C. Fisher, E. A. Fitzgerald, M. Flechl, I. Fleck, P. Fleischmann, S. Fleischmann, G. T. Fletcher, G. Fletcher, T. Flick, A. Floderus, L. R. Flores Castillo, A. C. Florez Bustos, M. J. Flowerdew, A. Formica, A. Forti, D. Fortin, D. Fournier, H. Fox, S. Fracchia, P. Francavilla, M. Franchini, S. Franchino, D. Francis, M. Franklin, S. Franz, M. Fraternali, S. T. French, C. Friedrich, F. Friedrich, D. Froidevaux, J. A. Frost, C. Fukunaga, E. Fullana Torregrosa, B. G. Fulsom, J. Fuster, C. Gabaldon, O. Gabizon, A. Gabrielli, A. Gabrielli, S. Gadatsch, S. Gadomski, G. Gagliardi, P. Gagnon, C. Galea, B. Galhardo, E. J. Gallas, V. Gallo, B. J. Gallop, P. Gallus, G. Galster, K. K. Gan, R. P. Gandrajula, J. Gao, Y. S. Gao, F. M. Garay Walls, F. Garberson, C. García, J. E. García Navarro, M. Garcia-Sciveres, R. W. Gardner, N. Garelli, V. Garonne, C. Gatti, G. Gaudio, B. Gaur, L. Gauthier, P. Gauzzi, I. L. Gavrilenko, C. Gay, G. Gaycken, E. N. Gazis, P. Ge, Z. Gecse, C. N. P. Gee, D. A. A. Geerts, Ch. Geich-Gimbel, K. Gellerstedt, C. Gemme, A. Gemmell, M. H. Genest, S. Gentile, M. George, S. George, D. Gerbaudo, A. Gershon, H. Ghazlane, N. Ghodbane, B. Giacobbe, S. Giagu, V. Giangiobbe, P. Giannetti, F. Gianotti, B. Gibbard, S. M. Gibson, M. Gilchriese, T. P. S. Gillam, D. Gillberg, G. Gilles, D. M. Gingrich, N. Giokaris, M. P. Giordani, R. Giordano, F. M. Giorgi, P. F. Giraud, D. Giugni, C. Giuliani, M. Giulini, B. K. Gjelsten, I. Gkialas, L. K. Gladilin, C. Glasman, J. Glatzer, P. C. F. Glaysher, A. Glazov, G. L. Glonti, M. Goblirsch-Kolb, J. R. Goddard, J. Godfrey, J. Godlewski, C. Goeringer, S. Goldfarb, T. Golling, D. Golubkov, A. Gomes, L. S. Gomez Fajardo, R. Gonçalo, J. Goncalves Pinto Firmino Da Costa, L. Gonella, S. González de la Hoz, G. Gonzalez Parra, M. L. Gonzalez Silva, S. Gonzalez-Sevilla, L. Goossens, P. A. Gorbounov, H. A. Gordon, I. Gorelov, G. Gorfine, B. Gorini, E. Gorini, A. Gorišek, E. Gornicki, A. T. Goshaw, C. Gössling, M. I. Gostkin, M. Gouighri, D. Goujdami, M. P. Goulette, A. G. Goussiou, C. Goy, S. Gozpinar, H. M. X. Grabas, L. Graber, I. Grabowska-Bold, P. Grafström, K.-J. Grahn, J. Gramling, E. Gramstad, S. Grancagnolo, V. Grassi, V. Gratchev, H. M. Gray, E. Graziani, O. G. Grebenyuk, Z. D. Greenwood, K. Gregersen, I. M. Gregor, P. Grenier, J. Griffiths, N. Grigalashvili, A. A. Grillo, K. Grimm, S. Grinstein, Ph. Gris, Y. V. Grishkevich, J.-F. Grivaz, J. P. Grohs, A. Grohsjean, E. Gross, J. Grosse-Knetter, G. C. Grossi, J. Groth-Jensen, Z. J. Grout, K. Grybel, L. Guan, F. Guescini, D. Guest, O. Gueta, C. Guicheney, E. Guido, T. Guillemin, S. Guindon, U. Gul, C. Gumpert, J. Gunther, J. Guo, S. Gupta, P. Gutierrez, N. G. Gutierrez Ortiz, C. Gutschow, N. Guttman, C. Guyot, C. Gwenlan, C. B. Gwilliam, A. Haas, C. Haber, H. K. Hadavand, N. Haddad, P. Haefner, S. Hageboeck, Z. Hajduk, H. Hakobyan, M. Haleem, D. Hall, G. Halladjian, K. Hamacher, P. Hamal, K. Hamano, M. Hamer, A. Hamilton, S. Hamilton, P. G. Hamnett, L. Han, K. Hanagaki, K. Hanawa, M. Hance, P. Hanke, J. R. Hansen, J. B. Hansen, J. D. Hansen, P. H. Hansen, K. Hara, A. S. Hard, T. Harenberg, S. Harkusha, D. Harper, R. D. Harrington, O. M. Harris, P. F. Harrison, F. Hartjes, S. Hasegawa, Y. Hasegawa, A Hasib, S. Hassani, S. Haug, M. Hauschild, R. Hauser, M. Havranek, C. M. Hawkes, R. J. Hawkings, A. D. Hawkins, T. Hayashi, D. Hayden, C. P. Hays, H. S. Hayward, S. J. Haywood, S. J. Head, T. Heck, V. Hedberg, L. Heelan, S. Heim, T. Heim, B. Heinemann, L. Heinrich, S. Heisterkamp, J. Hejbal, L. Helary, C. Heller, M. Heller, S. Hellman, D. Hellmich, C. Helsens, J. Henderson, R. C. W. Henderson, C. Hengler, A. Henrichs, A. M. Henriques Correia, S. Henrot-Versille, C. Hensel, G. H. Herbert, Y. Hernández Jiménez, R. Herrberg-Schubert, G. Herten, R. Hertenberger, L. Hervas, G. G. Hesketh, N. P. Hessey, R. Hickling, E. Higón-Rodriguez, E. Hill, J. C. Hill, K. H. Hiller, S. Hillert, S. J. Hillier, I. Hinchliffe, E. Hines, M. Hirose, D. Hirschbuehl, J. Hobbs, N. Hod, M. C. Hodgkinson, P. Hodgson, A. Hoecker, M. R. Hoeferkamp, J. Hoffman, D. Hoffmann, J. I. Hofmann, M. Hohlfeld, T. R. Holmes, T. M. Hong, L. Hooft van Huysduynen, J.-Y. Hostachy, S. Hou, A. Hoummada, J. Howard, J. Howarth, M. Hrabovsky, I. Hristova, J. Hrivnac, T. Hryn’ova, P. J. Hsu, S.-C. Hsu, D. Hu, X. Hu, Y. Huang, Z. Hubacek, F. Hubaut, F. Huegging, T. B. Huffman, E. W. Hughes, G. Hughes, M. Huhtinen, T. A. Hülsing, M. Hurwitz, N. Huseynov, J. Huston, J. Huth, G. Iacobucci, G. Iakovidis, I. Ibragimov, L. Iconomidou-Fayard, J. Idarraga, E. Ideal, P. Iengo, O. Igonkina, T. Iizawa, Y. Ikegami, K. Ikematsu, M. Ikeno, D. Iliadis, N. Ilic, Y. Inamaru, T. Ince, P. Ioannou, M. Iodice, K. Iordanidou, V. Ippolito, A. Irles Quiles, C. Isaksson, M. Ishino, M. Ishitsuka, R. Ishmukhametov, C. Issever, S. Istin, J. M. Iturbe Ponce, J. Ivarsson, A. V. Ivashin, W. Iwanski, H. Iwasaki, J. M. Izen, V. Izzo, B. Jackson, J. N. Jackson, M. Jackson, P. Jackson, M. R. Jaekel, V. Jain, K. Jakobs, S. Jakobsen, T. Jakoubek, J. Jakubek, D. O. Jamin, D. K. Jana, E. Jansen, H. Jansen, J. Janssen, M. Janus, G. Jarlskog, N. Javadov, T. Javůrek, L. Jeanty, G. -Y. Jeng, D. Jennens, P. Jenni, J. Jentzsch, C. Jeske, S. Jézéquel, H. Ji, W. Ji, J. Jia, Y. Jiang, M. Jimenez Belenguer, S. Jin, A. Jinaru, O. Jinnouchi, M. D. Joergensen, K. E. Johansson, P. Johansson, K. A. Johns, K. Jon-And, G. Jones, R. W. L. Jones, T. J. Jones, J. Jongmanns, P. M. Jorge, K. D. Joshi, J. Jovicevic, X. Ju, C. A. Jung, R. M. Jungst, P. Jussel, A. Juste Rozas, M. Kaci, A. Kaczmarska, M. Kado, H. Kagan, M. Kagan, E. Kajomovitz, S. Kama, N. Kanaya, M. Kaneda, S. Kaneti, T. Kanno, V. A. Kantserov, J. Kanzaki, B. Kaplan, A. Kapliy, D. Kar, K. Karakostas, N. Karastathis, M. Karnevskiy, S. N. Karpov, K. Karthik, V. Kartvelishvili, A. N. Karyukhin, L. Kashif, G. Kasieczka, R. D. Kass, A. Kastanas, Y. Kataoka, A. Katre, J. Katzy, V. Kaushik, K. Kawagoe, T. Kawamoto, G. Kawamura, S. Kazama, V. F. Kazanin, M. Y. Kazarinov, R. Keeler, P. T. Keener, R. Kehoe, M. Keil, J. S. Keller, H. Keoshkerian, O. Kepka, B. P. Kerševan, S. Kersten, K. Kessoku, J. Keung, F. Khalil-zada, H. Khandanyan, A. Khanov, A. Khodinov, A. Khomich, T. J. Khoo, G. Khoriauli, A. Khoroshilov, V. Khovanskiy, E. Khramov, J. Khubua, H. Y. Kim, H. Kim, S. H. Kim, N. Kimura, O. Kind, B. T. King, M. King, R. S. B. King, S. B. King, J. Kirk, A. E. Kiryunin, T. Kishimoto, D. Kisielewska, F. Kiss, T. Kitamura, T. Kittelmann, K. Kiuchi, E. Kladiva, M. Klein, U. Klein, K. Kleinknecht, P. Klimek, A. Klimentov, R. Klingenberg, J. A. Klinger, T. Klioutchnikova, P. F. Klok, E.-E. Kluge, P. Kluit, S. Kluth, E. Kneringer, E. B. F. G. Knoops, A. Knue, T. Kobayashi, M. Kobel, M. Kocian, P. Kodys, P. Koevesarki, T. Koffas, E. Koffeman, L. A. Kogan, S. Kohlmann, Z. Kohout, T. Kohriki, T. Koi, H. Kolanoski, I. Koletsou, J. Koll, A. A. Komar, Y. Komori, T. Kondo, N. Kondrashova, K. Köneke, A. C. König, S. König, T. Kono, R. Konoplich, N. Konstantinidis, R. Kopeliansky, S. Koperny, L. Köpke, A. K. Kopp, K. Korcyl, K. Kordas, A. Korn, A. A. Korol, I. Korolkov, E. V. Korolkova, V. A. Korotkov, O. Kortner, S. Kortner, V. V. Kostyukhin, S. Kotov, V. M. Kotov, A. Kotwal, C. Kourkoumelis, V. Kouskoura, A. Koutsman, R. Kowalewski, T. Z. Kowalski, W. Kozanecki, A. S. Kozhin, V. Kral, V. A. Kramarenko, G. Kramberger, D. Krasnopevtsev, M. W. Krasny, A. Krasznahorkay, J. K. Kraus, A. Kravchenko, S. Kreiss, M. Kretz, J. Kretzschmar, K. Kreutzfeldt, P. Krieger, K. Kroeninger, H. Kroha, J. Kroll, J. Kroseberg, J. Krstic, U. Kruchonak, H. Krüger, T. Kruker, N. Krumnack, Z. V. Krumshteyn, A. Kruse, M. C. Kruse, M. Kruskal, T. Kubota, S. Kuday, S. Kuehn, A. Kugel, A. Kuhl, T. Kuhl, V. Kukhtin, Y. Kulchitsky, S. Kuleshov, M. Kuna, J. Kunkle, A. Kupco, H. Kurashige, Y. A. Kurochkin, R. Kurumida, V. Kus, E. S. Kuwertz, M. Kuze, J. Kvita, A. La Rosa, L. La Rotonda, C. Lacasta, F. Lacava, J. Lacey, H. Lacker, D. Lacour, V. R. Lacuesta, E. Ladygin, R. Lafaye, B. Laforge, T. Lagouri, S. Lai, H. Laier, L. Lambourne, S. Lammers, C. L. Lampen, W. Lampl, E. Lançon, U. Landgraf, M. P. J. Landon, V. S. Lang, C. Lange, A. J. Lankford, F. Lanni, K. Lantzsch, S. Laplace, C. Lapoire, J. F. Laporte, T. Lari, M. Lassnig, P. Laurelli, W. Lavrijsen, A. T. Law, P. Laycock, B. T. Le, O. Le Dortz, E. Le Guirriec, E. Le Menedeu, T. LeCompte, F. Ledroit-Guillon, C. A. Lee, H. Lee, J. S. H. Lee, S. C. Lee, L. Lee, G. Lefebvre, M. Lefebvre, F. Legger, C. Leggett, A. Lehan, M. Lehmacher, G. Lehmann Miotto, X. Lei, A. G. Leister, M. A. L. Leite, R. Leitner, D. Lellouch, B. Lemmer, K. J. C. Leney, T. Lenz, G. Lenzen, B. Lenzi, R. Leone, K. Leonhardt, S. Leontsinis, C. Leroy, C. G. Lester, C. M. Lester, M. Levchenko, J. Levêque, D. Levin, L. J. Levinson, M. Levy, A. Lewis, G. H. Lewis, A. M. Leyko, M. Leyton, B. Li, B. Li, H. Li, H. L. Li, L. Li, S. Li, Y. Li, Z. Liang, H. Liao, B. Liberti, P. Lichard, K. Lie, J. Liebal, W. Liebig, C. Limbach, A. Limosani, M. Limper, S. C. Lin, F. Linde, B. E. Lindquist, J. T. Linnemann, E. Lipeles, A. Lipniacka, M. Lisovyi, T. M. Liss, D. Lissauer, A. Lister, A. M. Litke, B. Liu, D. Liu, J. B. Liu, K. Liu, L. Liu, M. Liu, M. Liu, Y. Liu, M. Livan, S. S. A. Livermore, A. Lleres, J. Llorente Merino, S. L. Lloyd, F. Lo Sterzo, E. Lobodzinska, P. Loch, W. S. Lockman, T. Loddenkoetter, F. K. Loebinger, A. E. Loevschall-Jensen, A. Loginov, C. W. Loh, T. Lohse, K. Lohwasser, M. Lokajicek, V. P. Lombardo, B. A. Long, J. D. Long, R. E. Long, L. Lopes, D. Lopez Mateos, B. Lopez Paredes, J. Lorenz, N. Lorenzo Martinez, M. Losada, P. Loscutoff, X. Lou, A. Lounis, J. Love, P. A. Love, A. J. Lowe, F. Lu, H. J. Lubatti, C. Luci, A. Lucotte, F. Luehring, W. Lukas, L. Luminari, O. Lundberg, B. Lund-Jensen, M. Lungwitz, D. Lynn, R. Lysak, E. Lytken, H. Ma, L. L. Ma, G. Maccarrone, A. Macchiolo, J. Machado Miguens, D. Macina, D. Madaffari, R. Madar, H. J. Maddocks, W. F. Mader, A. Madsen, M. Maeno, T. Maeno, E. Magradze, K. Mahboubi, J. Mahlstedt, S. Mahmoud, C. Maiani, C. Maidantchik, A. Maio, S. Majewski, Y. Makida, N. Makovec, P. Mal, B. Malaescu, Pa. Malecki, V. P. Maleev, F. Malek, U. Mallik, D. Malon, C. Malone, S. Maltezos, V. M. Malyshev, S. Malyukov, J. Mamuzic, B. Mandelli, L. Mandelli, I. Mandić, R. Mandrysch, J. Maneira, A. Manfredini, L. Manhaes de Andrade Filho, J. A. Manjarres Ramos, A. Mann, P. M. Manning, A. Manousakis-Katsikakis, B. Mansoulie, R. Mantifel, L. Mapelli, L. March, J. F. Marchand, G. Marchiori, M. Marcisovsky, C. P. Marino, C. N. Marques, F. Marroquim, S. P. Marsden, Z. Marshall, L. F. Marti, S. Marti-Garcia, B. Martin, B. Martin, J. P. Martin, T. A. Martin, V. J. Martin, B. Martin dit Latour, H. Martinez, M. Martinez, S. Martin-Haugh, A. C. Martyniuk, M. Marx, F. Marzano, A. Marzin, L. Masetti, T. Mashimo, R. Mashinistov, J. Masik, A. L. Maslennikov, I. Massa, N. Massol, P. Mastrandrea, A. Mastroberardino, T. Masubuchi, P. Matricon, H. Matsunaga, T. Matsushita, P. Mättig, S. Mättig, J. Mattmann, J. Maurer, S. J. Maxfield, D. A. Maximov, R. Mazini, L. Mazzaferro, G. Mc Goldrick, S. P. Mc Kee, A. McCarn, R. L. McCarthy, T. G. McCarthy, N. A. McCubbin, K. W. McFarlane, J. A. Mcfayden, G. Mchedlidze, T. Mclaughlan, S. J. McMahon, R. A. McPherson, A. Meade, J. Mechnich, M. Medinnis, S. Meehan, S. Mehlhase, A. Mehta, K. Meier, C. Meineck, B. Meirose, C. Melachrinos, B. R. Mellado Garcia, F. Meloni, A. Mengarelli, S. Menke, E. Meoni, K. M. Mercurio, S. Mergelmeyer, N. Meric, P. Mermod, L. Merola, C. Meroni, F. S. Merritt, H. Merritt, A. Messina, J. Metcalfe, A. S. Mete, C. Meyer, C. Meyer, J-P. Meyer, J. Meyer, R. P. Middleton, S. Migas, L. Mijović, G. Mikenberg, M. Mikestikova, M. Mikuž, D. W. Miller, C. Mills, A. Milov, D. A. Milstead, D. Milstein, A. A. Minaenko, M. Miñano Moya, I. A. Minashvili, A. I. Mincer, B. Mindur, M. Mineev, Y. Ming, L. M. Mir, G. Mirabelli, T. Mitani, J. Mitrevski, V. A. Mitsou, S. Mitsui, A. Miucci, P. S. Miyagawa, J. U. Mjörnmark, T. Moa, K. Mochizuki, V. Moeller, S. Mohapatra, W. Mohr, S. Molander, R. Moles-Valls, K. Mönig, C. Monini, J. Monk, E. Monnier, J. Montejo Berlingen, F. Monticelli, S. Monzani, R. W. Moore, A. Moraes, N. Morange, J. Morel, D. Moreno, M. Moreno Llácer, P. Morettini, M. Morgenstern, M. Morii, S. Moritz, A. K. Morley, G. Mornacchi, J. D. Morris, L. Morvaj, H. G. Moser, M. Mosidze, J. Moss, R. Mount, E. Mountricha, S. V. Mouraviev, E. J. W. Moyse, S. Muanza, R. D. Mudd, F. Mueller, J. Mueller, K. Mueller, T. Mueller, T. Mueller, D. Muenstermann, Y. Munwes, J. A. Murillo Quijada, W. J. Murray, H. Musheghyan, E. Musto, A. G. Myagkov, M. Myska, O. Nackenhorst, J. Nadal, K. Nagai, R. Nagai, Y. Nagai, K. Nagano, A. Nagarkar, Y. Nagasaka, M. Nagel, A. M. Nairz, Y. Nakahama, K. Nakamura, T. Nakamura, I. Nakano, H. Namasivayam, G. Nanava, R. Narayan, T. Nattermann, T. Naumann, G. Navarro, R. Nayyar, H. A. Neal, P. Yu. Nechaeva, T. J. Neep, A. Negri, G. Negri, M. Negrini, S. Nektarijevic, A. Nelson, T. K. Nelson, S. Nemecek, P. Nemethy, A. A. Nepomuceno, M. Nessi, M. S. Neubauer, M. Neumann, R. M. Neves, P. Nevski, F. M. Newcomer, P. R. Newman, D. H. Nguyen, R. B. Nickerson, R. Nicolaidou, B. Nicquevert, J. Nielsen, N. Nikiforou, A. Nikiforov, V. Nikolaenko, I. Nikolic-Audit, K. Nikolics, K. Nikolopoulos, P. Nilsson, Y. Ninomiya, A. Nisati, R. Nisius, T. Nobe, L. Nodulman, M. Nomachi, I. Nomidis, S. Norberg, M. Nordberg, J. Novakova, S. Nowak, M. Nozaki, L. Nozka, K. Ntekas, G. Nunes Hanninger, T. Nunnemann, E. Nurse, F. Nuti, B. J. O’Brien, F. O’grady, D. C. O’Neil, V. O’Shea, F. G. Oakham, H. Oberlack, T. Obermann, J. Ocariz, A. Ochi, M. I. Ochoa, S. Oda, S. Odaka, H. Ogren, A. Oh, S. H. Oh, C. C. Ohm, H. Ohman, T. Ohshima, W. Okamura, H. Okawa, Y. Okumura, T. Okuyama, A. Olariu, A. G. Olchevski, S. A. Olivares Pino, D. Oliveira Damazio, E. Oliver Garcia, A. Olszewski, J. Olszowska, A. Onofre, P. U. E. Onyisi, C. J. Oram, M. J. Oreglia, Y. Oren, D. Orestano, N. Orlando, C. Oropeza Barrera, R. S. Orr, B. Osculati, R. Ospanov, G. Otero y Garzon, H. Otono, M. Ouchrif, E. A. Ouellette, F. Ould-Saada, A. Ouraou, K. P. Oussoren, Q. Ouyang, A. Ovcharova, M. Owen, V. E. Ozcan, N. Ozturk, K. Pachal, A. Pacheco Pages, C. Padilla Aranda, M. Pagáčová, S. Pagan Griso, E. Paganis, C. Pahl, F. Paige, P. Pais, K. Pajchel, G. Palacino, S. Palestini, D. Pallin, A. Palma, J. D. Palmer, Y. B. Pan, E. Panagiotopoulou, J. G. Panduro Vazquez, P. Pani, N. Panikashvili, S. Panitkin, D. Pantea, L. Paolozzi, Th. D. Papadopoulou, K. Papageorgiou, A. Paramonov, D. Paredes Hernandez, M. A. Parker, F. Parodi, J. A. Parsons, U. Parzefall, E. Pasqualucci, S. Passaggio, A. Passeri, F. Pastore, Fr. Pastore, G. Pásztor, S. Pataraia, N. D. Patel, J. R. Pater, S. Patricelli, T. Pauly, J. Pearce, M. Pedersen, S. Pedraza Lopez, R. Pedro, S. V. Peleganchuk, D. Pelikan, H. Peng, B. Penning, J. Penwell, D. V. Perepelitsa, E. Perez Codina, M. T. Pérez García-Estañ, V. Perez Reale, L. Perini, H. Pernegger, R. Perrino, R. Peschke, V. D. Peshekhonov, K. Peters, R. F. Y. Peters, B. A. Petersen, J. Petersen, T. C. Petersen, E. Petit, A. Petridis, C. Petridou, E. Petrolo, F. Petrucci, M. Petteni, N. E. Pettersson, R. Pezoa, P. W. Phillips, G. Piacquadio, E. Pianori, A. Picazio, E. Piccaro, M. Piccinini, R. Piegaia, D. T. Pignotti, J. E. Pilcher, A. D. Pilkington, J. Pina, M. Pinamonti, A. Pinder, J. L. Pinfold, A. Pingel, B. Pinto, S. Pires, M. Pitt, C. Pizio, M.-A. Pleier, V. Pleskot, E. Plotnikova, P. Plucinski, S. Poddar, F. Podlyski, R. Poettgen, L. Poggioli, D. Pohl, M. Pohl, G. Polesello, A. Policicchio, R. Polifka, A. Polini, C. S. Pollard, V. Polychronakos, K. Pommès, L. Pontecorvo, B. G. Pope, G. A. Popeneciu, D. S. Popovic, A. Poppleton, X. Portell Bueso, G. E. Pospelov, S. Pospisil, K. Potamianos, I. N. Potrap, C. J. Potter, C. T. Potter, G. Poulard, J. Poveda, V. Pozdnyakov, P. Pralavorio, A. Pranko, S. Prasad, R. Pravahan, S. Prell, D. Price, J. Price, L. E. Price, D. Prieur, M. Primavera, M. Proissl, K. Prokofiev, F. Prokoshin, E. Protopapadaki, S. Protopopescu, J. Proudfoot, M. Przybycien, H. Przysiezniak, E. Ptacek, E. Pueschel, D. Puldon, M. Purohit, P. Puzo, J. Qian, G. Qin, Y. Qin, A. Quadt, D. R. Quarrie, W. B. Quayle, D. Quilty, A. Qureshi, V. Radeka, V. Radescu, S. K. Radhakrishnan, P. Radloff, P. Rados, F. Ragusa, G. Rahal, S. Rajagopalan, M. Rammensee, A. S. Randle-Conde, C. Rangel-Smith, K. Rao, F. Rauscher, T. C. Rave, T. Ravenscroft, M. Raymond, A. L. Read, D. M. Rebuzzi, A. Redelbach, G. Redlinger, R. Reece, K. Reeves, L. Rehnisch, A. Reinsch, H. Reisin, M. Relich, C. Rembser, Z. L. Ren, A. Renaud, M. Rescigno, S. Resconi, B. Resende, P. Reznicek, R. Rezvani, R. Richter, M. Ridel, P. Rieck, M. Rijssenbeek, A. Rimoldi, L. Rinaldi, E. Ritsch, I. Riu, F. Rizatdinova, E. Rizvi, S. H. Robertson, A. Robichaud-Veronneau, D. Robinson, J. E. M. Robinson, A. Robson, C. Roda, L. Rodrigues, S. Roe, O. Røhne, S. Rolli, A. Romaniouk, M. Romano, G. Romeo, E. Romero Adam, N. Rompotis, L. Roos, E. Ros, S. Rosati, K. Rosbach, M. Rose, P. L. Rosendahl, O. Rosenthal, V. Rossetti, E. Rossi, L. P. Rossi, R. Rosten, M. Rotaru, I. Roth, J. Rothberg, D. Rousseau, C. R. Royon, A. Rozanov, Y. Rozen, X. Ruan, F. Rubbo, I. Rubinskiy, V. I. Rud, C. Rudolph, M. S. Rudolph, F. Rühr, A. Ruiz-Martinez, Z. Rurikova, N. A. Rusakovich, A. Ruschke, J. P. Rutherfoord, N. Ruthmann, Y. F. Ryabov, M. Rybar, G. Rybkin, N. C. Ryder, A. F. Saavedra, S. Sacerdoti, A. Saddique, I. Sadeh, H. F-W. Sadrozinski, R. Sadykov, F. Safai Tehrani, H. Sakamoto, Y. Sakurai, G. Salamanna, A. Salamon, M. Saleem, D. Salek, P. H. Sales De Bruin, D. Salihagic, A. Salnikov, J. Salt, B. M. Salvachua Ferrando, D. Salvatore, F. Salvatore, A. Salvucci, A. Salzburger, D. Sampsonidis, A. Sanchez, J. Sánchez, V. Sanchez Martinez, H. Sandaker, R. L. Sandbach, H. G. Sander, M. P. Sanders, M. Sandhoff, T. Sandoval, C. Sandoval, R. Sandstroem, D. P. C. Sankey, A. Sansoni, C. Santoni, R. Santonico, H. Santos, I. Santoyo Castillo, K. Sapp, A. Sapronov, J. G. Saraiva, B. Sarrazin, G. Sartisohn, O. Sasaki, Y. Sasaki, I. Satsounkevitch, G. Sauvage, E. Sauvan, P. Savard, D. O. Savu, C. Sawyer, L. Sawyer, D. H. Saxon, J. Saxon, C. Sbarra, A. Sbrizzi, T. Scanlon, D. A. Scannicchio, M. Scarcella, J. Schaarschmidt, P. Schacht, D. Schaefer, R. Schaefer, S. Schaepe, S. Schaetzel, U. Schäfer, A. C. Schaffer, D. Schaile, R. D. Schamberger, V. Scharf, V. A. Schegelsky, D. Scheirich, M. Schernau, M. I. Scherzer, C. Schiavi, J. Schieck, C. Schillo, M. Schioppa, S. Schlenker, E. Schmidt, K. Schmieden, C. Schmitt, C. Schmitt, S. Schmitt, B. Schneider, Y. J. Schnellbach, U. Schnoor, L. Schoeffel, A. Schoening, B. D. Schoenrock, A. L. S. Schorlemmer, M. Schott, D. Schouten, J. Schovancova, M. Schram, S. Schramm, M. Schreyer, C. Schroeder, N. Schuh, M. J. Schultens, H.-C. Schultz-Coulon, H. Schulz, M. Schumacher, B. A. Schumm, Ph. Schune, A. Schwartzman, Ph. Schwegler, Ph. Schwemling, R. Schwienhorst, J. Schwindling, T. Schwindt, M. Schwoerer, F. G. Sciacca, E. Scifo, G. Sciolla, W. G. Scott, F. Scuri, F. Scutti, J. Searcy, G. Sedov, E. Sedykh, S. C. Seidel, A. Seiden, F. Seifert, J. M. Seixas, G. Sekhniaidze, S. J. Sekula, K. E. Selbach, D. M. Seliverstov, G. Sellers, N. Semprini-Cesari, C. Serfon, L. Serin, L. Serkin, T. Serre, R. Seuster, H. Severini, F. Sforza, A. Sfyrla, E. Shabalina, M. Shamim, L. Y. Shan, J. T. Shank, Q. T. Shao, M. Shapiro, P. B. Shatalov, K. Shaw, P. Sherwood, S. Shimizu, C. O. Shimmin, M. Shimojima, T. Shin, M. Shiyakova, A. Shmeleva, M. J. Shochet, D. Short, S. Shrestha, E. Shulga, M. A. Shupe, S. Shushkevich, P. Sicho, D. Sidorov, A. Sidoti, F. Siegert, Dj. Sijacki, O. Silbert, J. Silva, Y. Silver, D. Silverstein, S. B. Silverstein, V. Simak, O. Simard, Lj. Simic, S. Simion, E. Simioni, B. Simmons, R. Simoniello, M. Simonyan, P. Sinervo, N. B. Sinev, V. Sipica, G. Siragusa, A. Sircar, A. N. Sisakyan, S. Yu. Sivoklokov, J. Sjölin, T. B. Sjursen, H. P. Skottowe, K. Yu. Skovpen, P. Skubic, M. Slater, T. Slavicek, K. Sliwa, V. Smakhtin, B. H. Smart, L. Smestad, S. Yu. Smirnov, Y. Smirnov, L. N. Smirnova, O. Smirnova, K. M. Smith, M. Smizanska, K. Smolek, A. A. Snesarev, G. Snidero, J. Snow, S. Snyder, R. Sobie, F. Socher, J. Sodomka, A. Soffer, D. A. Soh, C. A. Solans, M. Solar, J. Solc, E. Yu. Soldatov, U. Soldevila, E. Solfaroli Camillocci, A. A. Solodkov, O. V. Solovyanov, V. Solovyev, P. Sommer, H. Y. Song, N. Soni, A. Sood, A. Sopczak, V. Sopko, B. Sopko, V. Sorin, M. Sosebee, R. Soualah, P. Soueid, A. M. Soukharev, D. South, S. Spagnolo, F. Spanò, W. R. Spearman, R. Spighi, G. Spigo, M. Spousta, T. Spreitzer, B. Spurlock, R. D. St. Denis, S. Staerz, J. Stahlman, R. Stamen, E. Stanecka, R. W. Stanek, C. Stanescu, M. Stanescu-Bellu, M. M. Stanitzki, S. Stapnes, E. A. Starchenko, J. Stark, P. Staroba, P. Starovoitov, R. Staszewski, P. Stavina, G. Steele, P. Steinberg, I. Stekl, B. Stelzer, H. J. Stelzer, O. Stelzer-Chilton, H. Stenzel, S. Stern, G. A. Stewart, J. A. Stillings, M. C. Stockton, M. Stoebe, G. Stoicea, P. Stolte, S. Stonjek, A. R. Stradling, A. Straessner, M. E. Stramaglia, J. Strandberg, S. Strandberg, A. Strandlie, E. Strauss, M. Strauss, P. Strizenec, R. Ströhmer, D. M. Strom, R. Stroynowski, S. A. Stucci, B. Stugu, N. A. Styles, D. Su, J. Su, HS. Subramania, R. Subramaniam, A. Succurro, Y. Sugaya, C. Suhr, M. Suk, V. V. Sulin, S. Sultansoy, T. Sumida, X. Sun, J. E. Sundermann, K. Suruliz, G. Susinno, M. R. Sutton, Y. Suzuki, M. Svatos, S. Swedish, M. Swiatlowski, I. Sykora, T. Sykora, D. Ta, K. Tackmann, J. Taenzer, A. Taffard, R. Tafirout, N. Taiblum, Y. Takahashi, H. Takai, R. Takashima, H. Takeda, T. Takeshita, Y. Takubo, M. Talby, A. A. Talyshev, J. Y. C. Tam, M. C. Tamsett, K. G. Tan, J. Tanaka, R. Tanaka, S. Tanaka, S. Tanaka, A. J. Tanasijczuk, K. Tani, N. Tannoury, S. Tapprogge, S. Tarem, F. Tarrade, G. F. Tartarelli, P. Tas, M. Tasevsky, T. Tashiro, E. Tassi, A. Tavares Delgado, Y. Tayalati, F. E. Taylor, G. N. Taylor, W. Taylor, F. A. Teischinger, M. Teixeira Dias Castanheira, P. Teixeira-Dias, K. K. Temming, H. Ten Kate, P. K. Teng, S. Terada, K. Terashi, J. Terron, S. Terzo, M. Testa, R. J. Teuscher, J. Therhaag, T. Theveneaux-Pelzer, S. Thoma, J. P. Thomas, J. Thomas-Wilsker, E. N. Thompson, P. D. Thompson, P. D. Thompson, A. S. Thompson, L. A. Thomsen, E. Thomson, M. Thomson, W. M. Thong, R. P. Thun, F. Tian, M. J. Tibbetts, V. O. Tikhomirov, Yu. A. Tikhonov, S. Timoshenko, E. Tiouchichine, P. Tipton, S. Tisserant, T. Todorov, S. Todorova-Nova, B. Toggerson, J. Tojo, S. Tokár, K. Tokushuku, K. Tollefson, L. Tomlinson, M. Tomoto, L. Tompkins, K. Toms, N. D. Topilin, E. Torrence, H. Torres, E. Torró Pastor, J. Toth, F. Touchard, D. R. Tovey, H. L. Tran, T. Trefzger, L. Tremblet, A. Tricoli, I. M. Trigger, S. Trincaz-Duvoid, M. F. Tripiana, N. Triplett, W. Trischuk, B. Trocmé, C. Troncon, M. Trottier-McDonald, M. Trovatelli, P. True, M. Trzebinski, A. Trzupek, C. Tsarouchas, J.C-L. Tseng, P. V. Tsiareshka, D. Tsionou, G. Tsipolitis, N. Tsirintanis, S. Tsiskaridze, V. Tsiskaridze, E. G. Tskhadadze, I. I. Tsukerman, V. Tsulaia, S. Tsuno, D. Tsybychev, A. Tudorache, V. Tudorache, A. N. Tuna, S. A. Tupputi, S. Turchikhin, D. Turecek, I. Turk Cakir, R. Turra, P. M. Tuts, A. Tykhonov, M. Tylmad, M. Tyndel, K. Uchida, I. Ueda, R. Ueno, M. Ughetto, M. Ugland, M. Uhlenbrock, F. Ukegawa, G. Unal, A. Undrus, G. Unel, F. C. Ungaro, Y. Unno, D. Urbaniec, P. Urquijo, G. Usai, A. Usanova, L. Vacavant, V. Vacek, B. Vachon, N. Valencic, S. Valentinetti, A. Valero, L. Valery, S. Valkar, E. Valladolid Gallego, S. Vallecorsa, J. A. Valls Ferrer, R. Van Berg, P. C. Van Der Deijl, R. van der Geer, H. van der Graaf, R. Van Der Leeuw, D. van der Ster, N. van Eldik, P. van Gemmeren, J. Van Nieuwkoop, I. van Vulpen, M. C. van Woerden, M. Vanadia, W. Vandelli, R. Vanguri, A. Vaniachine, P. Vankov, F. Vannucci, G. Vardanyan, R. Vari, E. W. Varnes, T. Varol, D. Varouchas, A. Vartapetian, K. E. Varvell, V. I. Vassilakopoulos, F. Vazeille, T. Vazquez Schroeder, J. Veatch, F. Veloso, S. Veneziano, A. Ventura, D. Ventura, M. Venturi, N. Venturi, A. Venturini, V. Vercesi, M. Verducci, W. Verkerke, J. C. Vermeulen, A. Vest, M. C. Vetterli, O. Viazlo, I. Vichou, T. Vickey, O. E. Vickey Boeriu, G. H. A. Viehhauser, S. Viel, R. Vigne, M. Villa, M. Villaplana Perez, E. Vilucchi, M. G. Vincter, V. B. Vinogradov, J. Virzi, I. Vivarelli, F. Vives Vaque, S. Vlachos, D. Vladoiu, M. Vlasak, A. Vogel, P. Vokac, G. Volpi, M. Volpi, H. von der Schmitt, H. von Radziewski, E. von Toerne, V. Vorobel, K. Vorobev, M. Vos, R. Voss, J. H. Vossebeld, N. Vranjes, M. Vranjes Milosavljevic, V. Vrba, M. Vreeswijk, T. Vu Anh, R. Vuillermet, I. Vukotic, Z. Vykydal, W. Wagner, P. Wagner, S. Wahrmund, J. Wakabayashi, J. Walder, R. Walker, W. Walkowiak, R. Wall, P. Waller, B. Walsh, C. Wang, C. Wang, F. Wang, H. Wang, H. Wang, J. Wang, J. Wang, K. Wang, R. Wang, S. M. Wang, T. Wang, X. Wang, C. Wanotayaroj, A. Warburton, C. P. Ward, D. R. Wardrope, M. Warsinsky, A. Washbrook, C. Wasicki, I. Watanabe, P. M. Watkins, A. T. Watson, I. J. Watson, M. F. Watson, G. Watts, S. Watts, B. M. Waugh, S. Webb, M. S. Weber, S. W. Weber, J. S. Webster, A. R. Weidberg, P. Weigell, B. Weinert, J. Weingarten, C. Weiser, H. Weits, P. S. Wells, T. Wenaus, D. Wendland, Z. Weng, T. Wengler, S. Wenig, N. Wermes, M. Werner, P. Werner, M. Wessels, J. Wetter, K. Whalen, A. White, M. J. White, R. White, S. White, D. Whiteson, D. Wicke, F. J. Wickens, W. Wiedenmann, M. Wielers, P. Wienemann, C. Wiglesworth, L. A. M. Wiik-Fuchs, P. A. Wijeratne, A. Wildauer, M. A. Wildt, H. G. Wilkens, J. Z. Will, H. H. Williams, S. Williams, C. Willis, S. Willocq, J. A. Wilson, A. Wilson, I. Wingerter-Seez, F. Winklmeier, M. Wittgen, T. Wittig, J. Wittkowski, S. J. Wollstadt, M. W. Wolter, H. Wolters, B. K. Wosiek, J. Wotschack, M. J. Woudstra, K. W. Wozniak, M. Wright, M. Wu, S. L. Wu, X. Wu, Y. Wu, E. Wulf, T. R. Wyatt, B. M. Wynne, S. Xella, M. Xiao, D. Xu, L. Xu, B. Yabsley, S. Yacoob, M. Yamada, H. Yamaguchi, Y. Yamaguchi, A. Yamamoto, K. Yamamoto, S. Yamamoto, T. Yamamura, T. Yamanaka, K. Yamauchi, Y. Yamazaki, Z. Yan, H. Yang, H. Yang, U. K. Yang, Y. Yang, S. Yanush, L. Yao, W.-M. Yao, Y. Yasu, E. Yatsenko, K. H. Yau Wong, J. Ye, S. Ye, A. L. Yen, E. Yildirim, M. Yilmaz, R. Yoosoofmiya, K. Yorita, R. Yoshida, K. Yoshihara, C. Young, C. J. S. Young, S. Youssef, D. R. Yu, J. Yu, J. M. Yu, J. Yu, L. Yuan, A. Yurkewicz, B. Zabinski, R. Zaidan, A. M. Zaitsev, A. Zaman, S. Zambito, L. Zanello, D. Zanzi, A. Zaytsev, C. Zeitnitz, M. Zeman, A. Zemla, K. Zengel, O. Zenin, T. Ženiš, D. Zerwas, G. Zevi della Porta, D. Zhang, F. Zhang, H. Zhang, J. Zhang, L. Zhang, X. Zhang, Z. Zhang, Z. Zhao, A. Zhemchugov, J. Zhong, B. Zhou, L. Zhou, N. Zhou, C. G. Zhu, H. Zhu, J. Zhu, Y. Zhu, X. Zhuang, A. Zibell, D. Zieminska, N. I. Zimine, C. Zimmermann, R. Zimmermann, S. Zimmermann, S. Zimmermann, Z. Zinonos, M. Ziolkowski, G. Zobernig, A. Zoccoli, M. zur Nedden, G. Zurzolo, V. Zutshi, L. Zwalinski

**Affiliations:** 1 Department of Physics, University of Adelaide, Adelaide, Australia; 2 Physics Department, SUNY Albany, Albany, NY USA; 3 Department of Physics, University of Alberta, Edmonton, AB Canada; 4 Department of Physics, Ankara University, Ankara, Turkey; 5 LAPP, CNRS/IN2P3 and Université de Savoie, Annecy-le-Vieux, France; 6 High Energy Physics Division, Argonne National Laboratory, Argonne, IL USA; 7 Department of Physics, University of Arizona, Tucson, AZ USA; 8 Department of Physics, The University of Texas at Arlington, Arlington, TX USA; 9 Physics Department, University of Athens, Athens, Greece; 10 Physics Department, National Technical University of Athens, Zografou, Greece; 11 Institute of Physics, Azerbaijan Academy of Sciences, Baku, Azerbaijan; 12 Institut de Física d’Altes Energies and Departament de Física de la Universitat Autònoma de Barcelona, Barcelona, Spain; 13 Institute of Physics, University of Belgrade, Belgrade, Serbia; 14 Department for Physics and Technology, University of Bergen, Bergen, Norway; 15 Physics Division, Lawrence Berkeley National Laboratory and University of California, Berkeley, CA USA; 16 Department of Physics, Humboldt University, Berlin, Germany; 17 Albert Einstein Center for Fundamental Physics and Laboratory for High Energy Physics, University of Bern, Bern, Switzerland; 18 School of Physics and Astronomy, University of Birmingham, Birmingham, UK; 19 Department of Physics, Bogazici University, Istanbul, Turkey; 20 INFN Sezione di Bologna, Bologna, Italy; 21 Physikalisches Institut, University of Bonn, Bonn, Germany; 22 Department of Physics, Boston University, Boston, MA USA; 23 Department of Physics, Brandeis University, Waltham, MA USA; 24 Universidade Federal do Rio De Janeiro COPPE/EE/IF, Rio de Janeiro, Brazil; 25 Physics Department, Brookhaven National Laboratory, Upton, NY USA; 26 National Institute of Physics and Nuclear Engineering, Bucharest, Romania; 27 Departamento de Física, Universidad de Buenos Aires, Buenos Aires, Argentina; 28 Cavendish Laboratory, University of Cambridge, Cambridge, UK; 29 Department of Physics, Carleton University, Ottawa, ON Canada; 30 CERN, Geneva, Switzerland; 31 Enrico Fermi Institute, University of Chicago, Chicago, IL USA; 32 Departamento de Física, Pontificia Universidad Católica de Chile, Santiago, Chile; 33 Institute of High Energy Physics, Chinese Academy of Sciences, Beijing, China; 34 Laboratoire de Physique Corpusculaire, Clermont Université and Université Blaise Pascal and CNRS/IN2P3, Clermont-Ferrand, France; 35 Nevis Laboratory, Columbia University, Irvington, NY USA; 36 Niels Bohr Institute, University of Copenhagen, Kobenhavn, Denmark; 37 INFN Gruppo Collegato di Cosenza, Laboratori Nazionali di Frascati, Frascati, Italy; 38 AGH University of Science and Technology, Faculty of Physics and Applied Computer Science, Kraków, Poland; 39 The Henryk Niewodniczanski Institute of Nuclear Physics, Polish Academy of Sciences, Kraków, Poland; 40 Physics Department, Southern Methodist University, Dallas, TX USA; 41 Physics Department, University of Texas at Dallas, Richardson, TX USA; 42 DESY, Hamburg and Zeuthen, Berlin, Germany; 43 Institut für Experimentelle Physik IV, Technische Universität Dortmund, Dortmund, Germany; 44 Institut für Kern- und Teilchenphysik, Technische Universität Dresden, Dresden, Germany; 45 Department of Physics, Duke University, Durham, NC USA; 46 SUPA, School of Physics and Astronomy, University of Edinburgh, Edinburgh, UK; 47 INFN Laboratori Nazionali di Frascati, Frascati, Italy; 48 Fakultät für Mathematik und Physik, Albert-Ludwigs-Universität, Freiburg, Germany; 49 Section de Physique, Université de Genève, Geneva, Switzerland; 50 INFN Sezione di Genova, Genoa, Italy; 51 E. Andronikashvili Institute of Physics, Iv. Javakhishvili Tbilisi State University, Tbilisi, Georgia; 52 II Physikalisches Institut, Justus-Liebig-Universität Giessen, Giessen, Germany; 53 SUPA, School of Physics and Astronomy, University of Glasgow, Glasgow, UK; 54 II Physikalisches Institut, Georg-August-Universität, Göttingen, Germany; 55 Laboratoire de Physique Subatomique et de Cosmologie, Université Grenoble-Alpes, CNRS/IN2P3 Grenoble, France; 56 Department of Physics, Hampton University, Hampton, VA USA; 57 Laboratory for Particle Physics and Cosmology, Harvard University, Cambridge, MA USA; 58 Kirchhoff-Institut für Physik, Ruprecht-Karls-Universität Heidelberg, Heidelberg, Germany; 59 Faculty of Applied Information Science, Hiroshima Institute of Technology, Hiroshima, Japan; 60 Department of Physics, Indiana University, Bloomington, IN USA; 61 Institut für Astro- und Teilchenphysik, Leopold-Franzens-Universität, Innsbruck, Austria; 62 University of Iowa, Iowa City, IA USA; 63 Department of Physics and Astronomy, Iowa State University, Ames, IA USA; 64 Joint Institute for Nuclear Research, JINR Dubna, Dubna, Russia; 65 KEK, High Energy Accelerator Research Organization, Tsukuba, Japan; 66 Graduate School of Science, Kobe University, Kobe, Japan; 67 Faculty of Science, Kyoto University, Kyoto, Japan; 68 Kyoto University of Education, Kyoto, Japan; 69 Department of Physics, Kyushu University, Fukuoka, Japan; 70 Instituto de Física La Plata, Universidad Nacional de La Plata and CONICET, La Plata, Argentina; 71 Physics Department, Lancaster University, Lancaster, UK; 72 INFN Sezione di Lecce, Lecce, Italy; 73 Oliver Lodge Laboratory, University of Liverpool, Liverpool, UK; 74 Department of Physics, Jožef Stefan Institute and University of Ljubljana, Ljubljana, Slovenia; 75 School of Physics and Astronomy, Queen Mary University of London, London, UK; 76 Department of Physics, Royal Holloway University of London, Surrey, UK; 77 Department of Physics and Astronomy, University College London, London, UK; 78 Louisiana Tech University, Ruston, LA USA; 79 Laboratoire de Physique Nucléaire et de Hautes Energies, UPMC and Université Paris-Diderot and CNRS/IN2P3, Paris, France; 80 Fysiska institutionen, Lunds universitet, Lund, Sweden; 81 Departamento de Fisica Teorica C-15, Universidad Autonoma de Madrid, Madrid, Spain; 82 Institut für Physik, Universität Mainz, Mainz, Germany; 83 School of Physics and Astronomy, University of Manchester, Manchester, UK; 84 CPPM, Aix-Marseille Université and CNRS/IN2P3, Marseille, France; 85 Department of Physics, University of Massachusetts, Amherst, MA USA; 86 Department of Physics, McGill University, Montreal, QC Canada; 87 School of Physics, University of Melbourne, Melbourne, VIC Australia; 88 Department of Physics, The University of Michigan, Ann Arbor, MI USA; 89 Department of Physics and Astronomy, Michigan State University, East Lansing, MI USA; 90 INFN Sezione di Milano, Milan, Italy; 91 B.I. Stepanov Institute of Physics, National Academy of Sciences of Belarus, Minsk, Republic of Belarus; 92 National Scientific and Educational Centre for Particle and High Energy Physics, Minsk, Republic of Belarus; 93 Department of Physics, Massachusetts Institute of Technology, Cambridge, MA USA; 94 Group of Particle Physics, University of Montreal, Montreal, QC Canada; 95 P.N. Lebedev Institute of Physics, Academy of Sciences, Moscow, Russia; 96 Institute for Theoretical and Experimental Physics (ITEP), Moscow, Russia; 97 Moscow Engineering and Physics Institute (MEPhI), Moscow, Russia; 98 D.V. Skobeltsyn Institute of Nuclear Physics, M.V. Lomonosov Moscow State University, Moscow, Russia; 99 Fakultät für Physik, Ludwig-Maximilians-Universität München, Munich, Germany; 100 Max-Planck-Institut für Physik (Werner-Heisenberg-Institut), Munich, Germany; 101 Nagasaki Institute of Applied Science, Nagasaki, Japan; 102 Graduate School of Science and Kobayashi-Maskawa Institute, Nagoya University, Nagoya, Japan; 103 INFN Sezione di Napoli, Naples, Italy; 104 Department of Physics and Astronomy, University of New Mexico, Albuquerque, NM USA; 105 Institute for Mathematics, Astrophysics and Particle Physics, Radboud University Nijmegen/Nikhef, Nijmegen, The Netherlands; 106 Nikhef National Institute for Subatomic Physics and University of Amsterdam, Amsterdam, The Netherlands; 107 Department of Physics, Northern Illinois University, DeKalb, IL USA; 108 Budker Institute of Nuclear Physics, SB RAS, Novosibirsk Russia; 109 Department of Physics, New York University, New York, NY USA; 110 Ohio State University, Columbus, OH USA; 111 Faculty of Science, Okayama University, Okayama, Japan; 112 Homer L. Dodge Department of Physics and Astronomy, University of Oklahoma, Norman, OK USA; 113 Department of Physics, Oklahoma State University, Stillwater, OK USA; 114 Palacký University, RCPTM, Olomouc Czech Republic; 115 Center for High Energy Physics, University of Oregon, Eugene, OR USA; 116 LAL, Université Paris-Sud and CNRS/IN2P3, Orsay, France; 117 Graduate School of Science, Osaka University, Osaka, Japan; 118 Department of Physics, University of Oslo, Oslo, Norway; 119 Department of Physics, Oxford University, Oxford, UK; 120 INFN Sezione di Pavia, Pavia, Italy; 121 Department of Physics, University of Pennsylvania, Philadelphia, PA USA; 122 Petersburg Nuclear Physics Institute, Gatchina, Russia; 123 INFN Sezione di Pisa, Pisa, Italy; 124 Department of Physics and Astronomy, University of Pittsburgh, Pittsburgh, PA USA; 125 Laboratorio de Instrumentacao e Fisica Experimental de Particulas, LIP, Lisbon, Portugal; 126 Institute of Physics, Academy of Sciences of the Czech Republic, Praha, Czech Republic; 127 Czech Technical University in Prague, Praha, Czech Republic; 128 Faculty of Mathematics and Physics, Charles University in Prague, Praha, Czech Republic; 129 State Research Center Institute for High Energy Physics, Protvino, Russia; 130 Particle Physics Department, Rutherford Appleton Laboratory, Didcot, UK; 131 Physics Department, University of Regina, Regina, SK Canada; 132 Ritsumeikan University, Kusatsu, Shiga Japan; 133 INFN Sezione di Roma, Rome, Italy; 134 INFN Sezione di Roma Tor Vergata, Rome, Italy; 135 INFN Sezione di Roma Tre, Rome, Italy; 136 Faculté des Sciences Ain Chock, Réseau Universitaire de Physique des Hautes Energies, Université Hassan II, Casablanca, Morocco; 137 DSM/IRFU (Institut de Recherches sur les Lois Fondamentales de l’Univers), CEA Saclay (Commissariat à l’Energie Atomique et aux Energies Alternatives), Gif-sur-Yvette, France; 138 Santa Cruz Institute for Particle Physics, University of California Santa Cruz, Santa Cruz, CA USA; 139 Department of Physics, University of Washington, Seattle, WA USA; 140 Department of Physics and Astronomy, University of Sheffield, Sheffield, UK; 141 Department of Physics, Shinshu University, Nagano, Japan; 142 Fachbereich Physik, Universität Siegen, Siegen, Germany; 143 Department of Physics, Simon Fraser University, Burnaby, BC Canada; 144 SLAC National Accelerator Laboratory, Stanford, CA USA; 145 Faculty of Mathematics, Physics and Informatics, Comenius University, Bratislava, Slovakia; 146 Department of Physics, University of Cape Town, Cape Town, South Africa; 147 Department of Physics, Stockholm University, Stockholm, Sweded; 148 Physics Department, Royal Institute of Technology, Stockholm, Sweden; 149 Departments of Physics and Astronomy and Chemistry, Stony Brook University, Stony Brook, NY USA; 150 Department of Physics and Astronomy, University of Sussex, Brighton, UK; 151 School of Physics, University of Sydney, Sydney, Australia; 152 Institute of Physics, Academia Sinica, Taipei, Taiwan; 153 Department of Physics, Technion: Israel Institute of Technology, Haifa, Israel; 154 Raymond and Beverly Sackler School of Physics and Astronomy, Tel Aviv University, Tel Aviv, Israel; 155 Department of Physics, Aristotle University of Thessaloniki, Thessaloniki, Greece; 156 International Center for Elementary Particle Physics and Department of Physics, The University of Tokyo, Tokyo, Japan; 157 Graduate School of Science and Technology, Tokyo Metropolitan University, Tokyo, Japan; 158 Department of Physics, Tokyo Institute of Technology, Tokyo, Japan; 159 Department of Physics, University of Toronto, Toronto, ON Canada; 160 TRIUMF, Vancouver, BC Canada; 161 Faculty of Pure and Applied Sciences, University of Tsukuba, Tsukuba, Japan; 162 Department of Physics and Astronomy, Tufts University, Medford, MA USA; 163 Centro de Investigaciones, Universidad Antonio Narino, Bogota, Colombia; 164 Department of Physics and Astronomy, University of California Irvine, Irvine, CA USA; 165 INFN Gruppo Collegato di Udine, Sezione di Trieste, Udine, Italy; 166 Department of Physics, University of Illinois, Urbana, IL USA; 167 Department of Physics and Astronomy, University of Uppsala, Uppsala, Sweden; 168 Instituto de Física Corpuscular (IFIC) and Departamento de Física Atómica, Molecular y Nuclear and Departamento de Ingeniería Electrónica and Instituto de Microelectrónica de Barcelona (IMB-CNM), University of Valencia and CSIC, Valencia, Spain; 169 Department of Physics, University of British Columbia, Vancouver, BC Canada; 170 Department of Physics and Astronomy, University of Victoria, Victoria, BC Canada; 171 Department of Physics, University of Warwick, Coventry, USA; 172 Waseda University, Tokyo, Japan; 173 Department of Particle Physics, The Weizmann Institute of Science, Rehovot, Israel; 174 Department of Physics, University of Wisconsin, Madison, WI USA; 175 Fakultät für Physik und Astronomie, Julius-Maximilians-Universität, Würzburg, Germany; 176 Fachbereich C Physik, Bergische Universität Wuppertal, Wuppertal, Germany; 177 Department of Physics, Yale University, New Haven, CT USA; 178 Yerevan Physics Institute, Yerevan, Armenia; 179 Centre de Calcul de l’Institut National de Physique Nucléaire et de Physique des Particules (IN2P3), Villeurbanne, France; 180 Department of Physics, Gazi University, Ankara, Turkey; 181 Division of Physics, TOBB University of Economics and Technology, Ankara, Turkey; 182 Turkish Atomic Energy Authority, Ankara, Turkey; 183 Vinca Institute of Nuclear Sciences, University of Belgrade, Belgrade, Serbia; 184 Department of Physics, Dogus University, Istanbul, Turkey; 185 Department of Physics Engineering, Gaziantep University, Gaziantep, Turkey; 186 Dipartimento di Fisica e Astronomia, Università di Bologna, Bologna, Italy; 187 Federal University of Juiz de Fora (UFJF), Juiz de Fora, Brazil; 188 Federal University of Sao Joao del Rei (UFSJ), Sao Joao del Rei, Brazil; 189 Instituto de Fisica, Universidade de Sao Paulo, Sao Paulo, Brazil; 190 National Institute for Research and Development of Isotopic and Molecular Technologies, Physics Department, Cluj Napoca, Romania; 191 University Politehnica Bucharest, Bucharest, Romania; 192 West University in Timisoara, Timisoara, Romania; 193 Departamento de Física, Universidad Técnica Federico Santa María, Valparaíso, Chile; 194 Department of Modern Physics, University of Science and Technology of China, Anhui, China; 195 Department of Physics, Nanjing University, Jiangsu, China; 196 School of Physics, Shandong University, Shandong, China; 197 Physics Department, Shanghai Jiao Tong University, Shanghai, China; 198 Dipartimento di Fisica, Università della Calabria, Rende, Italy; 199 Marian Smoluchowski Institute of Physics, Jagiellonian University, Kraków, Poland; 200 Dipartimento di Fisica, Università di Genova, Genoa, Italy; 201 High Energy Physics Institute, Tbilisi State University, Tbilisi, Georgia; 202 Physikalisches Institut, Ruprecht-Karls-Universität Heidelberg, Heidelberg, Germany; 203 ZITI Institut für technische Informatik, Ruprecht-Karls-Universität Heidelberg, Mannheim, Germany; 204 Dipartimento di Matematica e Fisica, Università del Salento, Lecce, Italy; 205 Dipartimento di Fisica, Università di Milano, Milan, Italy; 206 Dipartimento di Fisica, Università di Napoli, Naples, Italy; 207 Dipartimento di Fisica, Università di Pavia, Pavia, Italy; 208 Dipartimento di Fisica E. Fermi, Università di Pisa, Pisa, Italy; 209 Faculdade de Ciências, Universidade de Lisboa, Lisbon, Portugal; 210 Department of Physics, University of Coimbra, Coimbra, Portugal; 211 Centro de Física Nuclear da Universidade de Lisboa, Lisbon, Portugal; 212 Departamento de Fisica, Universidade do Minho, Braga, Portugal; 213 Departamento de Fisica Teorica y del Cosmos and CAFPE, Universidad de Granada, Granada, Spain; 214 Dep Fisica and CEFITEC of Faculdade de Ciencias e Tecnologia, Universidade Nova de Lisboa, Caparica, Portugal; 215 Dipartimento di Fisica, Sapienza Università di Roma, Rome, Italy; 216 Dipartimento di Fisica, Università di Roma Tor Vergata, Rome, Italy; 217 Dipartimento di Matematica e Fisica, Università Roma Tre, Rome, Italy; 218 Centre National de l’Energie des Sciences Techniques Nucleaires, Rabat, Morocco; 219 Faculté des Sciences Semlalia, Université Cadi Ayyad, LPHEA-Marrakech, Marrakesh, Morocco; 220 Faculté des Sciences, Université Mohamed Premier and LPTPM, Oujda, Morocco; 221 Faculté des sciences, Université Mohammed V-Agdal, Rabat, Morocco; 222 Department of Subnuclear Physics, Institute of Experimental Physics of the Slovak Academy of Sciences, Kosice, Slovak Republic; 223 Department of Physics, University of Johannesburg, Johannesburg, South Africa; 224 School of Physics, University of the Witwatersrand, Johannesburg, South Africa; 225 The Oskar Klein Centre, Stockholm, Sweden; 226 Department of Physics and Astronomy, York University, Toronto, ON Canada; 227 ICTP, Trieste, Italy; 228 Dipartimento di Chimica, Fisica e Ambiente, Università di Udine, Udine, Italy; 229 CERN, 1211 Geneva 23, Switzerland

## Abstract

A search is presented for direct top squark pair production using events with at least two leptons including a same-flavour opposite-sign pair with invariant mass consistent with the $Z$ boson mass, jets tagged as originating from $b$-quarks and missing transverse momentum. The analysis is performed with proton–proton collision data at $\sqrt{s}=8{\hbox {\ TeV}}$ collected with the ATLAS detector at the LHC in 2012 corresponding to an integrated luminosity of 20.3 fb$^{-1}$. No excess beyond the Standard Model expectation is observed. Interpretations of the results are provided in models based on the direct pair production of the heavier top squark state ($\tilde{t}_2$) followed by the decay to the lighter top squark state ($\tilde{t}_1$) via $\tilde{t}_2 \rightarrow Z\tilde{t}_1$, and for $\tilde{t}_1$ pair production in natural gauge-mediated supersymmetry breaking scenarios where the neutralino ($\tilde{\chi }^0_1$) is the next-to-lightest supersymmetric particle and decays producing a $Z$ boson and a gravitino ($\tilde{G}$) via the $\tilde{\chi }^0_1 \rightarrow Z\tilde{G}$ process.

## Introduction

Supersymmetry (SUSY) [1–9] is an extension of the Standard Model (SM) which predicts new bosonic partners for the existing fermions and fermionic partners for the known bosons. In the framework of a generic $R$-parity conserving minimal supersymmetric extension of the SM (MSSM) [10–14], SUSY particles are produced in pairs and the lightest supersymmetric particle (LSP) is stable, providing a possible dark matter candidate.

In a large variety of models, the LSP is the lightest neutralino ($\tilde{\chi }_{1}^{0}$) which is a mixture of the neutral supersymmetric partners of the gauge and Higgs bosons, known as gauginos and higgsinos. Similarly, charginos are a mixture of the charged gauginos and higgsinos, with the lightest denoted by $\tilde{\chi }_{1}^{\pm }$. The scalar partners of right-handed and left-handed quarks, $\tilde{q}_R$ and $\tilde{q}_L$, mix to form two mass eigenstates, $\tilde{q}_1$ and $\tilde{q}_2$, with $\tilde{q}_1$ defined to be the lighter of the two. Naturalness arguments [15, 16] imply that the supersymmetric partners of the top quark (stops) are light, with mass below $1{\hbox {\ TeV}}$.

Searches for direct pair production of the $\tilde{t}_1$ have been performed by the ATLAS [17–22] and CMS [23–26] collaborations. These searches with $\tilde{t}_{1}\rightarrow t\tilde{\chi }_{1}^{0}$ currently have little sensitivity to scenarios where the lightest stop is only slightly heavier than the sum of the masses of the top quark and the LSP, due to the similarities in kinematics with SM top pair production ($t\bar{t}$). In those scenarios, by considering instead the direct pair production of the heavy stop ($\tilde{t}^{}_{2}$) decaying via $\tilde{t}_{2}\rightarrow Z \tilde{t}_{1}$, stop signals can be discriminated from the $t\bar{t}$ background by requiring a same-flavour opposite-sign (SFOS) lepton pair originating from the $Z$ boson decay. Requiring a third lepton, that in signal events can be produced from the top quark in the $\tilde{t}_{1}\rightarrow t\tilde{\chi }_{1}^{0}$ decay, can further reject $t\bar{t}$. Sensitivity to direct $\tilde{t}_{2}$ pair production can be obtained with this three-lepton signature even in models where additional decay modes of the $\tilde{t}_{2}$, such as $\tilde{t}_{2}\rightarrow t\tilde{\chi }_{1}^{0}$ or via the lightest Higgs boson ($h$) in $\tilde{t}_{2}\rightarrow h\tilde{t}_{1}$, are significant.

A similar signature can also occur in $\tilde{t}^{}_{1}$ pair production in gauge-mediated SUSY breaking (GMSB) models [27–32]. The $\tilde{\chi }_{1}^{0}$ from $\tilde{t}_{1}$ decay is typically the next-to-lightest supersymmetric particle (NLSP) and the supersymmetric partner of the graviton (gravitino, $\tilde{G}$) is typically the LSP and is very light ($m_{\tilde{G}} < 1 {\hbox {\ keV}}$). Assuming a mass scale of the messengers responsible for the supersymmetry breaking of around $10{\hbox {\ TeV}}$ and little fine tuning [15], the lightest stop is expected to have a mass of less than $400{\hbox {\ GeV}}$ [33]. The $\tilde{\chi }_{1}^{0}$ decays to either a $\gamma$, $Z$, or $h$ boson and a $\tilde{G}$. If the $\tilde{\chi }_{1}^{0}$ is higgsino-like, as suggested by naturalness arguments, it dominantly decays either via $\tilde{\chi }_{1}^{0}\rightarrow h\tilde{G}$ or via $\tilde{\chi }_{1}^{0}\rightarrow Z\tilde{G}$, in the latter case giving a $Z$ boson at the end of the stop decay chain.

In this paper a search for stop pair production is reported in final states characterised by the presence of a $Z$ boson with or without additional leptons, plus jets originating from $b$-quarks ($b$-jets) produced in the stop decay chain and significant missing transverse momentum from the undetected LSPs. Results are interpreted in simplified models featuring $\tilde{t}_2$ production and in the framework of natural GMSB. This paper presents the first result on $\tilde{t}_2$ direct pair production and extends the results of a previous ATLAS analysis, carried out using $7{\hbox {\ TeV}}$ data corresponding to an integrated luminosity of 2.05 fb$^{-1}$ [34], that excluded stop masses up to $310{\hbox {\ GeV}}$ for $115{\hbox {\ GeV}}<m_{\tilde{\chi }_{1}^{0}}<230{\hbox {\ GeV}}$ in natural GMSB scenarios.

## The ATLAS detector

ATLAS [35] is a general-purpose particle physics experiment at the LHC. The layout of the detector consists of inner tracking devices surrounded by a superconducting solenoid, electromagnetic and hadronic calorimeters and a muon spectrometer with a magnetic field produced by three large superconducting toroids each with eight coils. The inner tracking detector is formed from silicon pixel and microstrip detectors, and a straw tube transition radiation tracker, and provides precision tracking of charged particles for pseudorapidity $|\eta |<2.5$.^1^ The calorimeter system, placed outside the solenoid, covers $|\eta |<4.9$ and is composed of electromagnetic and hadronic sampling calorimeters with either liquid argon or scintillating tiles as the active medium. The muon spectrometer surrounds the calorimeter and consists of a system of precision tracking chambers within $|\eta |<2.7$, and detectors for triggering within $|\eta |<2.4$.

## Signal and background simulation

Monte Carlo (MC) simulated event samples are used to aid in the estimation of the SM background and to model the SUSY signal. MC samples are processed through a detector simulation [36] based on Geant4 [37] or a fast simulation using a parameterisation of the performance of the electromagnetic and hadronic calorimeters and Geant4 for the other parts of the detector [38], and are reconstructed in the same manner as the data. The simulation includes the effect of multiple $pp$ collisions in the same and neighbouring bunch crossings and is weighted to reproduce the observed distribution of the average number of collisions per bunch crossing. All MC samples used in the analysis are produced using the ATLAS underlying event tune 2B [39] unless otherwise stated.

The top-quark pair production background is simulated with Powheg Box r2129 [40–42] interfaced to Pythia 6.427 [43] for the fragmentation and hadronisation processes. The mass of the top quark is fixed at 172.5 GeV, and the next-to-leading order (NLO) parton distribution function (PDF) set CT10 [44] is used. The total cross section is calculated at next-to-next-to-leading-order (NNLO) including resummation of next-to-next-to-leading logarithmic (NNLL) soft gluon terms with top++2.0 [45–50]. The P2011C [51] MC tune is used for this sample. Samples generated with Alpgen 2.14 [52] interfaced with Herwig 6.510 [53], including Jimmy 4.3 [54] for the underlying event description, are used to evaluate generator systematic uncertainties, while Powheg Box r2129 interfaced to Herwig 6.510 and AcerMC 3.8 [55] interfaced to Pythia 6.426 are used for hadronisation and initial/final state radiation (ISR/FSR) uncertainty estimation respectively. Production of a single top quark in association with a $W$ boson is simulated with Powheg Box r2129 interfaced to Pythia 6.426 using the diagram removal scheme [56]. The nominal samples describing $t\bar{t}$ production in association with gauge bosons ($t\bar{t}V$) as well as single top production in association with a $Z$ boson ($tZ$) in the $t$- and $s$-channels, and the $tWZ$ process, are generated using the leading-order (LO) generator MadGraph5 1.3.33 [57] interfaced to Pythia 6.426 for the fragmentation and the hadronisation. The total cross sections of $t\bar{t}W$ and $t\bar{t}Z$ are normalised to NLO [58] while $tZ$ is normalised to the LO cross section from the generator, since NLO calculations are currently only available for the $t$-channel [59]. To estimate generator and hadronisation systematic uncertainties for the $t\bar{t}W$ and $t\bar{t}Z$ processes, Alpgen 2.14 interfaced with Herwig 6.520, including Jimmy 4.3, is used. Samples of $Z/\gamma ^*$ production in association with up to five jets are produced with Sherpa 1.4.1 [60] where $b$- and $c$-quarks are treated as massive. MC samples of dibosons ($ZZ$, $WZ$ and $WW$) decaying to final states with 2, 3 and 4 leptons are generated using Powheg Box r2129 interfaced to Pythia 8.163 [61]. Samples generated with aMC@NLO [62] (in MadGraph5 2.0.0.beta) interfaced to Pythia 6.427 or Herwig 6.510 are used to evaluate generator, hadronisation and scale variation uncertainties. Samples of tribosons ($WWW$, $ZWW$ and $ZZZ$) are generated with MadGraph5 1.3.33 interfaced to Pythia 6.426 and normalised to NLO [63]. Higgs boson production in association with a vector boson or $t\bar{t}$ pair is simulated with Pythia 8.165, with cross sections calculated at NNLO QCD + NLO electroweak precision, except $pp\rightarrow t\bar{t}h$, which is calculated at NLO QCD precision [64]. The multijet and $\gamma$+jet processes are simulated with Pythia 8.165 and Pythia 8.160 respectively.

Signal events are generated according to SUSY models using Herwig++ 2.5.2 [65] with the CTEQ6L1 PDF set. Signal cross sections are calculated at NLO + NLL accuracy [66–68]. The nominal cross section and the uncertainty are taken from an envelope of cross section predictions using different PDF sets and factorisation and renormalisation scales, as described in Ref. [69].

Direct $\tilde{t}^{}_{2}$ pair production is studied using a simplified model, where all SUSY particles are decoupled except for the $\tilde{t}_{2}$, $\tilde{t}_{1}$ and $\tilde{\chi }_{1}^{0}$, assumed to be the LSP. The only decays included in this model are $\tilde{t}^{}_{2}\rightarrow Z\tilde{t}^{}_{1}$ and $\tilde{t}^{}_{1}\rightarrow t\tilde{\chi }_1^{0}$. The mass of the top quark is fixed at 172.5 GeV. The mass difference between the lighter stop and the neutralino is set to $180{\hbox {\ GeV}}$, a region not excluded by previous searches [21], and signal samples are generated varying the masses of the $\tilde{t}^{}_{2}$ and $\tilde{\chi }_1^{0}$. In addition, dedicated samples also including the $\tilde{t}^{}_{2}\rightarrow h\tilde{t}^{}_{1}$ and $\tilde{t}^{}_{2}\rightarrow t\tilde{\chi }_1^{0}$ decay modes are used to interpret the results as a function of the $\tilde{t}^{}_{2}$ branching ratios. Simulated samples corresponding to direct $\tilde{t}^{}_{1}$ pair production for values of $m_{\tilde{t}^{}_{1}}=m_{\tilde{\chi }_1^{0}}+180{\hbox {\ GeV}}$ are also used in the analysis.

For the natural GMSB scenario, a very similar model to that of Ref. [34] is considered, with the Higgs boson assumed to be SM-like and with the mass set at $126{\hbox {\ GeV}}$, in agreement with the observation of a Higgs boson at the LHC [70, 71], and with $\tan \beta$, the ratio of the vacuum expectation value of the two neutral Higgs doublets of the MSSM, set to 5. The masses of the first and second generation squarks and gluinos (superpartners of the gluons) are above $5{\hbox {\ TeV}}$, and maximal mixing between the squark eigenstates is assumed for $\tilde{t}_{1}$. Only $\tilde{t}_{1}$ pair production is considered. $\tilde{\chi }_{1}^{0}$, $\tilde{\chi }_{2}^{0}$ and $\tilde{\chi }_{1}^{\pm }$ are assumed to be predominantly higgsino states. Hence, if $\tilde{\chi }_{2}^{0}$ or $\tilde{\chi }_{1}^{\pm }$ are produced in a decay chain, they decay to $\tilde{\chi }_{1}^{0}$ promptly with soft accompanying fermions. The branching fractions of the $\tilde{t}_{1}$ and higgsino decays are predicted by the model. If $m_{\tilde{t}_{1}}<m_{t}+m_{\tilde{\chi }_{1}^{0}}$, $\tilde{t}_{1}$ decays via $\tilde{t}_{1}\rightarrow b\tilde{\chi }_{1}^{\pm }$ exclusively, while if $m_{\tilde{t}_{1}}>m_{t}+m_{\tilde{\chi }_{1}^{0}}$, $\tilde{t}_{1}$ may also decay with similar probability via $\tilde{t}_{1}\rightarrow t\tilde{\chi }_{1}^{0}$ (or $t\tilde{\chi }_{2}^{0}$). For the model parameters considered, the $\tilde{\chi }_{1}^{0}$ predominantly decays to $Z\tilde{G}$ with branching ratios typically above 70 %. Signal samples are generated varying the $\tilde{t}_1$ and $\tilde{\chi }_1^{0}$ masses.

## Object identification and event selection

After the application of beam, detector and data quality requirements, the total luminosity considered in this analysis corresponds to 20.3 fb$^{-1}$. The uncertainty on the integrated luminosity is $\pm$2.8 %. It is derived, following the same methodology as that detailed in Ref. [72], from a preliminary calibration of the luminosity scale derived from beam-separation scans performed in November 2012.

Events are selected if they pass the single electron or muon triggers; these are fully efficient for lepton $p_{\mathrm{T}}>25{\hbox {\ GeV}}$. The presence of at least one primary vertex, with at least five tracks with $p_{\mathrm{T}}>0.4{\hbox {\ GeV}}$ associated to it, is required. In order to optimize the analysis and to perform data-driven background estimations, two categories of jets, electrons, muons and photons are defined: “candidate” and “signal” (with tighter selection criteria).

Jets are reconstructed from three-dimensional calorimeter energy clusters by using the anti-$k_t$ algorithm [73] with a radius parameter of 0.4. Jet energies are corrected [74] for detector inhomogeneities, the non-compensating nature of the calorimeter, and the impact of multiple overlapping *pp* interactions, using factors derived from test beam, cosmic ray and *pp* collision data and from a detailed Geant4 detector simulation. Events with any jet that fails the jet quality criteria designed to remove noise and non-collision backgrounds [74] are rejected. Jet candidates are required to have $p_{\mathrm{T}}>20{\hbox {\ GeV}}$ and $|\eta |<2.8$. Jets labelled as signal jets are further required to have $p_{\mathrm{T}}>30{\hbox {\ GeV}}$ and, for those with $p_{\mathrm{T}}<50{\hbox {\ GeV}}$ and $|\eta |<2.4$, the jet vertex fraction, defined as the fraction of the sum of the $p_{\mathrm{T}}$ of the tracks associated with the jet and matched to the selected primary vertex, normalised by the sum of the $p_{\mathrm{T}}$ of all tracks associated with the jet, is required to be larger than 25 %.

Identification of jets containing $b$-quarks ($b$-tagging) is performed with a dedicated algorithm based on a neural-network approach which uses the output weights of several $b$-tagging algorithms [75] as input. A requirement is chosen corresponding to a 60 % average efficiency obtained for $b$-jets in simulated $t\bar{t}$ events. The rejection factors for mis-tagging light quark jets, $c$-quark jets and $\tau$ leptons in simulated SM $t\bar{t}$ events are approximately 600, 8 and 24, respectively. Signal jets with $|\eta |<2.5$ which satisfy this *b-*tagging requirement are identified as *b-*jets. To compensate for differences between data and MC simulation in the $b$-tagging efficiencies and mis-tag rates, correction factors derived from different methods, such as the use of the $p_{\mathrm{T}}$ of muons relative to the axis of the jet [76] and a dedicated study in $t\bar{t}$ dominated regions [77], are applied to the simulated samples. A sample of $D^{*+}$ mesons is used for mis-tag rates of *c-*jets [78] and inclusive jet samples for mis-tag rates of a jet which does not originate from a *b-* or *c-*quark [79].

Electron candidates must satisfy the “medium” selection criteria described in Ref. [80], re-optimised for 2012 data, and are required to fulfil $p_{\mathrm{T}}> 10{\hbox {\ GeV}}$ and $\vert \eta \vert < 2.47$. Signal electrons must pass the previous requirements and also need to be isolated, i.e. the scalar sum of the $p_{\mathrm{T}}$ of charged-particle tracks within a cone of radius $\Delta R= 0.3$ around the candidate excluding its own track must be less than 16 % of the electron $p_{\mathrm{T}}$. In addition, a longitudinal impact parameter requirement of $\vert z_0 \sin \theta \vert <0.4~\mathrm{mm}$ is applied to signal electrons. The track parameter $z_{0}$ is defined with respect to the reconstructed primary vertex.

Muon candidates are required to have $p_{\mathrm{T}}> 10{\hbox {\ GeV}}$, $\vert \eta \vert < 2.4$ and are identified by matching an extrapolated inner detector track and one or more track segments in the muon spectrometer [81]. Signal muons are then required to be isolated, i.e. the scalar sum of the $p_{\mathrm{T}}$ of charged-particle tracks within a cone of radius $\Delta R = 0.3$ around the muon candidate excluding its own track must be less than 12 % of the muon $p_{\mathrm{T}}$. In addition, a longitudinal impact parameter requirement of $\vert z_0 \sin \theta \vert <0.4~\mathrm{mm}$ is applied to signal muons.

A signal lepton with $p_{\mathrm{T}}$ larger than $25{\hbox {\ GeV}}$ is required to match the one that triggered the event such that the efficiency of the trigger is $p_{\mathrm{T}}$ independent. The MC events are corrected to account for minor differences in the lepton trigger, reconstruction and identification efficiencies between data and MC simulation [80, 81].

To resolve ambiguities between reconstructed jets and leptons, jet candidates within a distance of $\Delta R=0.2$ of an electron candidate are rejected. Any electron or muon candidate within a distance of $\Delta R=0.4$ of any remaining jet candidate is also rejected. To suppress the rare case where two distinct tracks are mistakenly associated with one calorimeter energy cluster forming two electron candidates, if two electron candidates are found within a distance $\Delta R= 0.1$, the one with smaller transverse momentum is rejected. Finally, to suppress muon bremsstrahlung leading to an incorrect measurement of the transverse momentum, if an electron candidate and a muon candidate are within $\Delta R=0.1$, both are rejected.

Photons are used only for the $Z$+jets estimation in the two-lepton signal regions described in Sect. 5 and the overlap removal between photons and jets described below is performed only in this case. Photon candidates are required to have $p_{\mathrm{T}}>25{\hbox {\ GeV}}$, $|\eta |<2.47$ and must satisfy the “tight” selection criteria described in Ref. [82]. Signal photons are further required to be isolated, i.e. the scalar sum of transverse energy deposition in the calorimeter observed within a cone of radius $\Delta R=0.4$ around the photon candidate excluding its own energy deposition in the calorimeter must be less than $4{\hbox {\ GeV}}$. To resolve overlaps between reconstructed jets and photons, jet candidates within a distance of $\Delta R=0.2$ of a photon candidate are rejected.

The calculation of the missing transverse momentum, where its magnitude is referred to as $E_\mathrm{T}^\mathrm{miss}$ [83], is based on the vector sum of the transverse momenta of all electron, muon and jet candidates, as well as photons with $p_{\mathrm{T}}>10$ GeV and calibrated calorimeter energy clusters with $|\eta | < 4.9$ not associated with these objects. Clusters associated with electrons, photons and jets make use of the calibrations of these objects. For jets, the calibration includes the pile-up correction described above, whilst the jet vertex fraction requirement is not considered when selecting jet candidates for computing the $E_\mathrm{T}^\mathrm{miss}$. Clusters not associated with these objects are calibrated using both calorimeter and tracker information [83].

**Fig. 1:**
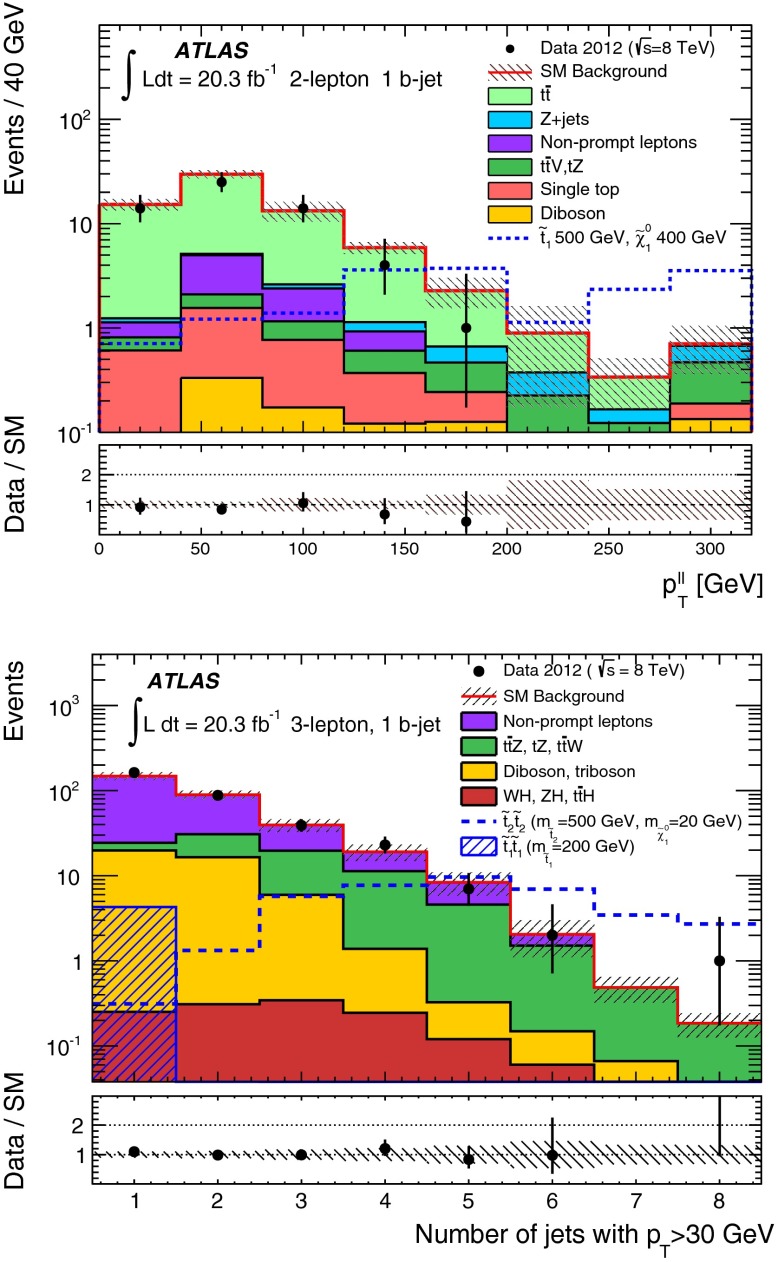
*Top* $p_{\mathrm{T}}(\ell \ell )$ distributions in SR2A before the $p_{\mathrm{T}}(\ell \ell )>80$ GeV and $\Delta \phi ^{\ell \ell }<1.5$ selections. *Bottom*, number of signal jets with $p_{\mathrm{T}}>30$ GeV in events with 3 signal leptons after the lepton, $m_{\ell \ell }$ and $b$-jets selections in SR3A. *Shaded bands* denote the background statistical and systematic uncertainty. For illustration, distributions for selected signal points are also shown: the stop natural GMSB model with $m_{\tilde{t}_{1}}=500{\hbox {\ GeV}}$, $m_{\tilde{\chi }_{1}^{0}}=400{\hbox {\ GeV}}$ (*top*) and the simplified model with $m_{\tilde{t}_{2}}=500{\hbox {\ GeV}}$, $m_{\tilde{t}_{1}}=200$ GeV and $m_{\tilde{\chi }^{0}_{1}}=20{\hbox {\ GeV}}$ for both direct $\tilde{t}^{}_{2}$ and $\tilde{t}^{}_{1}$ pair production (*bottom*). The last bin includes the histogram overflow

Five signal regions (SRs) are defined in the analysis aiming at final states with a $Z$ boson, $b$-jets, significant $E_\mathrm{T}^\mathrm{miss}$ and possibly additional leptons, as summarised in Table 1. They are characterised by the number of leptons (electrons or muons) required in the final state. For the two-lepton SRs (indicated as SR2A, SR2B and SR2C), events with exactly two leptons are selected, with the $p_{\mathrm{T}}$ of the leading one required to be larger than $25{\hbox {\ GeV}}$. They are required to be signal leptons and form a SFOS pair with invariant mass ($m_{\ell \ell }$) within $5{\hbox {\ GeV}}$ or $10{\hbox {\ GeV}}$ of the $Z$-boson mass. At least one *b-*jet is required. SR2A and SR2B are optimised for the small $m_{\tilde{t}_{1}}-m_{\tilde{\chi }_{1}^{0}}$ region of the natural GMSB model where low jet multiplicity is expected, whilst SR2C is optimised for the large $m_{\tilde{t}_{1}}-m_{\tilde{\chi }_{1}^{0}}$ region where the jet multiplicity is high. SR2A is optimised for a stop mass around $400{\hbox {\ GeV}}$ and SR2B is for $600{\hbox {\ GeV}}$. Since the $Z$ boson produced in stop signal events is typically boosted, the transverse momentum of the dilepton system, $p_{\mathrm{T}}(\ell \ell )$, tends to be high while the azimuthal separation $\Delta \phi ^{\ell \ell }$ tends to be low. This is illustrated by Fig. 1, which shows the $p_{\mathrm{T}}(\ell \ell )$ distribution after the lepton, $m_{\ell \ell }$, jet and $b$-jet requirements in SR2A are applied. Requirements of $\Delta \phi ^{\ell \ell }$ below 1.5 and $p_{\mathrm{T}}(\ell \ell )>80{\hbox {\ GeV}}$ or $160{\hbox {\ GeV}}$ are therefore applied in the SRs. Finally, to enhance the signal contribution, typically with large $E_\mathrm{T}^\mathrm{miss}$ due to the LSPs, $E_\mathrm{T}^\mathrm{miss}>160 {\hbox {\ GeV}}$ or $200 {\hbox {\ GeV}}$ is required depending on the targeted stop mass.

**Table 1:** Summary of the event selection in the signal and $t\bar{t}$ background control regions used in the analysis. The variables used are the number of leptons ($N^\mathrm{leptons}$), the $p_{\mathrm{T}}$ of the leading lepton ($p_{\mathrm{T}}(\ell _1)$), the dilepton flavour (SF: same-flavour; DF: different flavour), the dilepton invariant mass ($m_{\ell \ell }$), the number of $b$-jets ($N^{b\mathrm{-jets}}$), the number of jets regardless of their flavour ($N^\mathrm{jets}$), the $p_{\mathrm{T}}$ of the leading jet ($p_{\mathrm{T}}(\mathrm{jet}_1)$), the $p_{\mathrm{T}}$ of the $N^{\mathrm{jets}}$-th jet required in each region ($p_{\mathrm{T}}(\mathrm{jet}_N)$), the missing transverse momentum ($E_\mathrm{T}^\mathrm{miss}$), the transverse momentum of the dilepton system ($p_{\mathrm{T}}({\ell \ell })$), and the angular separation in the transverse plane between the leptons forming the SFOS pair ($\Delta \phi ^{\ell \ell }$)

	SR2A	SR2B	SR2C	CR2A	CR2C	SR3A	SR3B
$N^\mathrm{leptons}$	2	2	2	2	2	3	3
$p_{\mathrm{T}}(\ell _1)$ (GeV)	$>$25	$>$25	$>$25	$>$25	$>$25	$>$40	$>$60
Dilepton flavour	SF	SF	SF	SF, DF	SF, DF	SF	SF
$|m_{\ell \ell }-m_Z |$ (GeV)	$<$5	$<$10	$<$5	$<$50	$<$50	$<$10	$<$10
				$>$10 (SF)	$>$10 (SF)		
$N^{\mathrm{b}\text {-}\mathrm{jets}}$	$\ge$1	$\ge$1	$\ge$1	$\ge$1	$\ge$1	$\ge$1	$\ge$1
$N^\mathrm{jets}$	$3$, $4$	$3$, $4$	$\ge$5	$3$, $4$	$\ge$5	$\ge$5	$\ge$5
$p_{\mathrm{T}}(\mathrm{jet}_1)$ (GeV)	$>$30	$>$30	$>$30	$>$30	$>$30	$>$50	$>$40
$p_{\mathrm{T}}(\mathrm{jet}_N)$ (GeV)	$>$30	$>$30	$>$30	$>$30	$>$30	$>$30	$>$40
$E_\mathrm{T}^\mathrm{miss}$(GeV)	$>$160	$>$200	$>$160	$>$160	$>$120	$>$60	$>$60
$p_{\mathrm{T}}({\ell \ell })$ (GeV)	$>$80	$>$160	$>$80	$>$80	$>$80	–	$>$75
$\Delta \phi ^{\ell \ell }$ (rad)	$<$1.5	$<$1.5	$<$1.5	$<$1.5	$<$1.5	–	–

In the three-lepton SRs (indicated as SR3A and SR3B), at least three signal leptons with two of them forming an SFOS pair with invariant mass which is within $10{\hbox {\ GeV}}$ of the $Z$ boson mass are required. Two regions are optimised to give good sensitivity in the direct $\tilde{t}^{}_{2}$ pair production model for different $\tilde{t}^{}_{2}- \tilde{t}^{}_{1}$ mass splittings. The SR3A is aimed at signal models with low mass splitting where the $Z$-boson is not boosted. The SR3B is optimised for high mass splitting where the $Z$-boson is boosted requiring a minimum $p_{\mathrm{T}}$ of the dilepton system of 75 GeV. A high-$p_{\mathrm{T}}$ leading lepton with a minimum $p_{\mathrm{T}}$ requirement of $40 {\hbox {\ GeV}}$ or $60 {\hbox {\ GeV}}$ for SR3A and SR3B respectively, and at least one *b-*jet are required to suppress the diboson background. The signal is expected to have higher jet multiplicity than the SM background, due to the presence of two top quarks and two $Z$ bosons. This is illustrated by Fig. 1, which shows the jet multiplicity distribution after the lepton, $m_{\ell \ell }$, and $b$-jet requirements in SR3A are applied. Therefore at least five jets are required to increase the signal sensitivity.

## Background estimation

Two main sources of background can be distinguished in this analysis: events containing at least one non-prompt or fake lepton (mainly production of multijets and $W$ boson in association with jets in the two-lepton SRs, and production of top pairs and $Z$ boson in association with jets in the three-lepton SRs) and events with two or three prompt leptons (mainly $Z$+jets and $t\bar{t}$ in the two-lepton SRs, and $t\bar{t}V$, $tZ$, diboson and triboson events in the three-lepton SRs).

### Background from fake or non-prompt leptons

Fake leptons can originate from a misidentified light flavour quark or gluon jet (referred to as light flavour). Non-prompt leptons can originate from a semileptonic decay of a hadron containing a $b$- or $c$-quark (referred to as heavy flavour), or an electron from a photon conversion. The contribution from fake and non-prompt leptons is estimated from data with a matrix method similar to that described in Refs. [84, 85]. In order to perform the matrix method, two types of lepton identification criteria are defined: “tight”, corresponding to the signal lepton criteria described in Sect. 4, and “loose”, corresponding to candidate leptons. To increase the available statistics, muons within a $0.2 < \Delta R < 0.4$ distance from jets are also considered as loose muons in the method if the scalar sum of $p_{\mathrm{T}}$ of charged-particle tracks within a cone of radius $\Delta R = 0.3$ around the muon candidate excluding its own track is less than 30 % of the muon $p_{\mathrm{T}}$. The matrix method relates the number of events containing fake or non-prompt leptons to the number of observed events with tight or loose leptons using the probability for loose prompt, fake or non-prompt leptons to pass the tight criteria. The probability for loose prompt leptons to pass the tight selection criteria is obtained using a $Z\rightarrow \ell \ell$ data sample and is modelled as a function of the lepton $p_{\mathrm{T}}$. The probability for loose non-prompt leptons to pass the tight selection criteria is determined from data separately for heavy flavour in a $b\bar{b}$ enriched sample and for photon conversions in a $Z\rightarrow \mu \mu \gamma$ sample. This probability is modelled as a function of $p_{\mathrm{T}}$ and $\eta$ for electrons and of $p_{\mathrm{T}}$ and the number of jets for muons. Simulation studies show that the contribution of fake leptons originating from a misidentified light flavour quark or gluon jet is negligible in all the signal and data control regions used for the background estimation. The probability for loose non-prompt electrons passing the tight selection is calculated according to the fraction of heavy flavour and photon conversion obtained in MC for the different regions.

For SRs with two leptons, relations are obtained for the observed event counts as a function of the number of events containing prompt and non-prompt leptons. These can be solved simultaneously to estimate the number of background events with two tight lepton candidates with at least one non-prompt lepton. In the three-lepton SRs, the background from non-prompt leptons is estimated as in the two-lepton case by considering the leading lepton to be prompt, which simulation studies show to be true in $>$99 % of the events, and applying the same estimation method to the second and third leading leptons in the event. The results of the estimations have been validated with data in regions with similar background composition obtained by reversing the $E_\mathrm{T}^\mathrm{miss}$ or jet multiplicity cuts used in the SRs.

### $t\bar{t}$ background in the two-lepton channel

The dominant background in the two-lepton signal regions comes from $t\bar{t}$. The background prediction is normalised to data in dedicated control regions (CRs), and then extrapolated to the SRs. The observed number of events in the CRs are used to derive $t\bar{t}$ estimates in each of the SRs via a profile likelihood method [86].

The CRs are designed to have kinematic selections as similar as possible to the corresponding SRs in order to minimize systematic uncertainties on the extrapolation of the background to the SR. The CRs use both dilepton events with the same flavour (SF) and different flavour (DF) with the following dilepton mass requirements: $10{\hbox {\ GeV}}<|m_{\ell \ell }-m_{Z}|<50{\hbox {\ GeV}}$ (SF), and $|m_{\ell \ell }-m_{Z}|<50{\hbox {\ GeV}}$ (DF). Except for lepton-flavour dependent systematic uncertainties, SF and DF events are treated in the same way. Apart from the $m_{\ell \ell }$ requirements the CR corresponding to SR2A/B (labelled CR2A) has exactly the same selections as SR2A, whereas the CR for SR2C (labelled CR2C) has a looser $E_\mathrm{T}^\mathrm{miss}$ selection than the SR to increase the number of events in the CR.

For the background estimation neglecting any possible signal contribution in the CRs, the fit takes as input the number of expected background events in each CR and SR taken from MC or data-driven estimations and the number of observed events in the CRs. For each SR, the free parameter is the overall normalisation of the $t\bar{t}$ process. Each uncertainty source is treated as a nuisance parameter in the fit, constrained with a Gaussian function taking into account the correlations between different background sources. The likelihood function is the product of Poisson probability functions describing the observed and expected number of events in the CRs, and the Gaussian constraints on the nuisance parameters. The contribution from all other non-constrained processes are set at the theoretical expectation, but are allowed to vary within their uncertainties. The fitting procedure maximises this likelihood by adjusting the free and nuisance parameters. For the signal models considered in this paper the contamination of the CRs by signal events is small (typically less than 10 %).

The expected and observed number of events in the control regions are shown in Table 2. The MC simulation before the fit overestimates the number of $t\bar{t}$ events observed in both of the CRs. This mis-modelling at high $t\bar{t}$ transverse momentum ($p_{\text {T},t\bar{t}}$) has been observed in previous ATLAS analyses [87].

**Table 2:** Background fit results and observed numbers of events in the $t\bar{t}$ control regions for the two-lepton channel. The uncertainty shown is the sum of the statistical and systematic uncertainties. Nominal MC expectations are given for comparison

	CR2A	CR2C
Data	152	101
Fitted total SM	$152\pm 13$	$101\pm 11$
Fitted $t\bar{t}$	$128 \pm 13$	$88\pm 11$
Fitted single top	$12\pm 4$	$4.4\pm 3.2$
Fitted $Z$+jets	$0.62\pm 0.04$	$0.75\pm 0.07$
Fitted diboson	$1.6\pm 1.4$	$0.5 \pm 0.4$
Fitted $t\bar{t}V,tZ$	$1.6 \pm 0.4$	$1.7\pm 0.5$
Fitted non-prompt	$7.4\pm 2.4$	$6.1\pm 1.9$
MC exp. total SM	176	146
MC exp. $t\bar{t}$	152	132
MC exp. single top	13	5.2
MC exp. $Z$+jets	0.62	0.75
MC exp. diboson	1.7	0.5
MC exp. $t\bar{t}V,tZ$	1.6	1.7
Data-driven non-prompt	7.4	6.1

### $Z$+jets background in the two-lepton channel

Background events from $Z$-boson production associated with jets typically contain fake $E_\mathrm{T}^\mathrm{miss}$ due to resolution effects in the jet momentum measurement. Due to the limited statistics and the difficulty of accurately reproducing fake $E_\mathrm{T}^\mathrm{miss}$ in MC simulations, a data-driven “jet smearing method” [88] is used to estimate this contribution in the high $E_\mathrm{T}^\mathrm{miss}$ tail. In this method, well-measured $Z$+jets events with low $E_\mathrm{T}^\mathrm{miss}$ are selected. By applying jet energy resolution smearing to these events a pseudo-data sample with fake $E_\mathrm{T}^\mathrm{miss}$ is generated. The pseudo-data sample is then normalised to data in the $E_\mathrm{T}^\mathrm{miss}<80{\hbox {\ GeV}}$ region, after subtracting other SM background sources estimated by MC for real two lepton events and by the data-driven method for events with non-prompt leptons. Their contribution is less than 10 %. The jet energy resolution smearing function ($p_{\mathrm{T}}^{\text {reco}}/p_{\mathrm{T}}^{\text {truth}}$) is initially obtained from multijet MC simulation, where $p_{\mathrm{T}}^{\text {reco}}$ is the transverse momentum of the reconstructed jet and $p_{\mathrm{T}}^{\text {truth}}$ is the transverse momentum of the jet constructed from stable truth particles excluding muons and neutrinos. Stable particles are defined as those with a lifetime of 10 ps or more in the laboratory frame. The function is corrected using $\gamma$+jet data events where the photon and the jet are balanced. These events are selected by a single photon trigger and require at least one signal photon and one baseline jet. To suppress soft radiation that would affect the $p_{\mathrm{T}}$ balance between the jet and the photon, the angle between the leading jet and the leading photon in the transverse plane is required to be larger than 2.9 rad, and the second-leading jet is required to have $p_{\mathrm{T}}$ of less than 20 % of the $p_{\mathrm{T}}$ of the photon. Using the $p_{\mathrm{T}}$ of the balanced photon as reference for that of the jet, the $p_{\mathrm{T}}$ response of jets is measured in data and MC. The jet energy resolution smearing function is then modified to match $p_{\mathrm{T}}$ response between data and MC. The method is validated by closure tests using MC simulation, and also using data in the $80{\hbox {\ GeV}}<E_\mathrm{T}^\mathrm{miss}<160{\hbox {\ GeV}}$ region.

### Other backgrounds

The estimation of other background processes producing two or three prompt leptons, such as diboson, triboson, $t\bar{t}V$, $tZ$ or $Wt$ production, is performed using the MC samples described in Sect. 3.

Since $t\bar{t}Z$ is the main background in the three-lepton SRs and has a topology very similar to a $\tilde{t}^{}_{2}\rightarrow Z\tilde{t}^{}_{1}$ signal, dedicated validation regions with an enhanced contribution from this background and orthogonal to the SRs are defined to verify the MC prediction in data. These regions are defined requiring at least three leptons and the same $m_{\ell \ell }$ and $b$-jet requirements as the SRs. In order to enhance the $t\bar{t}Z$ contribution and reduce the possible contamination from signal events, the events are required to have from three to five jets with $p_{\mathrm{T}}>30$ GeV and fewer than five jets with $p_{\mathrm{T}}>50$ GeV. The $E_\mathrm{T}^\mathrm{miss}$ is required to be less than 150 GeV except for events with 5 jets with $p_{\mathrm{T}}>30$ GeV where the $E_\mathrm{T}^\mathrm{miss}$ is required to be less than 60 GeV to avoid overlaps with the SRs. The third leading lepton is required to have $p_{\mathrm{T}}>20$ GeV to reduce the contribution from non-prompt leptons. Two separate validation regions are defined using the $p_{\mathrm{T}}(\ell \ell )$ variable: VR3A with $p_{\mathrm{T}}(\ell \ell )<120$ GeV and VR3B with $p_{\mathrm{T}}(\ell \ell )>120$ GeV. The contamination from a potential signal can be large in these validation regions but would typically affect VR3A and VR3B differently depending on the $\tilde{t}^{}_{2}$-$\tilde{t}^{}_{1}$ mass splitting. Table 3 shows the expected number of events in these validation regions taken from MC or data-driven estimations together with the observed number of events. The expected contribution from selected signal models is also shown. The $t\bar{t}Z$ contribution is 40–50 % of the total expected event count, and a good agreement with data is observed in both regions.

**Table 3:** Number of events in the VR3A and VR3B $t\bar{t}Z$ validation regions together with the expectation for some signal points in the $\tilde{t}^{}_{2}$ simplified model. The errors on the backgrounds include both statistical and systematic uncertainties. Only statistical uncertainties are shown for the signal points

	VR3A	VR3B
Data	24	13
Total SM	19 $\pm$ 5	12.1 $\pm$ 3.2
MC exp. $t\bar{t}Z$	7.9 $\pm$ 2.1	5.9 $\pm$ 1.6
MC exp. $tZ$	2.7 $\pm$ 2.7	1.5 $\pm$ 1.5
Data-driven non-prompt	5.9 $\pm$ 2.9	2.7 $\pm$ 1.4
MC exp. diboson, triboson	1.5 $\pm$ 0.5	1.9 $\pm$ 0.6
MC exp. $t\bar{t}W$	0.35 $\pm$ 0.10	0.05 $\pm$ 0.02
MC exp. $Wh$, $Zh$, $t\bar{t}h$	0.3 $\pm$ 0.3	0.05 $\pm$ 0.05
($m_{\tilde{t}^{}_{2}},m_{\tilde{\chi }_1^0})=(500,20)$ GeV	1.6 $\pm$ 0.6	7.5 $\pm$ 1.2
($m_{\tilde{t}^{}_{2}},m_{\tilde{\chi }_1^0})=(500,120)$ GeV	3.3 $\pm$ 0.8	3.9 $\pm$ 0.8
($m_{\tilde{t}^{}_{2}},m_{\tilde{\chi }_1^0})=(550,20)$ GeV	0.6 $\pm$ 0.3	4.6 $\pm$ 0.7
($m_{\tilde{t}^{}_{2}},m_{\tilde{\chi }_1^0})=(550,220)$ GeV	2.7 $\pm$ 0.5	2.2 $\pm$ 0.5

## Systematic uncertainties

The dominant detector-related systematic effects are due to the jet energy scale (JES) and resolution (JER) uncertainties, and the uncertainties on the $b$-tagging efficiency and mistag rates.

The JES uncertainty is derived from a combination of simulation, test-beam data and in-situ measurements [74]. Additional terms accounting for flavour composition, flavour response, pile-up and *b-*jet scale uncertainties are taken into account. These uncertainties sum to 10–20 % of the total number of estimated background events depending on the SR. JER uncertainties are determined with an in-situ measurement of the jet response asymmetry in dijet events [89], and the impact on the SRs ranges between 1–10 %. Uncertainties associated with the $b$-tagging efficiency and mis-tagging of a $c$- and light-quark jet are obtained from the same techniques used in the derivation of their correction factors. The uncertainty on the expected number of background events in the SR due to $b$-tagging ranges between 4–10 %.

For the non-prompt lepton background estimation, uncertainties are assigned due to the statistical uncertainty on the number of data events with loose and tight leptons and due to the MC uncertainty on the relative composition of non-prompt electrons (heavy flavour and conversions). The uncertainties on the probabilities for loose leptons to pass the tight selections typically range between 10–45 %, are estimated by using alternative samples for their computation, and include possible dependencies on the lepton $p_{\mathrm{T}}$, $\eta$ or jet multiplicity. The overall impact of the non-prompt lepton background uncertainties on the expected number of background events are below 2 % in the 2-lepton SRs and approximately 15 % in the 3-lepton SRs.

The uncertainties on the MC modelling of background processes are determined by testing different generators as well as parton shower and hadronisation models. The systematic uncertainties on the modelling of $t\bar{t}$+jets, used only to determine the transfer factors between control and signal regions in the two-lepton case, are evaluated by comparing results obtained with the Powheg and Alpgen generators. The hadronisation uncertainty is addressed by comparing Powheg interfaced to Pythia6 with Powheg interfaced to Herwig+Jimmy. The uncertainty related to the amount of ISR/FSR is estimated using the predictions of dedicated AcerMC samples generated with different tuning parameters. The uncertainties on $t\bar{t}$ are dominated by these theoretical uncertainties after the fit. A 22 % cross section uncertainty is assumed for $t\bar{t}Z$ and $t\bar{t}W$ [58]. The uncertainties on the modelling of $t\bar{t}V$ are evaluated by comparing MadGraph interfaced to Pythia6 with Alpgen interfaced with Herwig+Jimmy. The uncertainty assigned on the diboson cross sections are 5 % for $ZZ$ [90] and 7 % for $WZ$ [91]. For diboson production processes, the uncertainties on the modelling are evaluated by comparing Powheg interfaced to Pythia8 with the aMC@NLO generator interfaced to Pythia6 and Herwig+Jimmy . For tribosons, $t\bar{t}h$ and $tZ$ production processes, which constitute a very small background in all signal regions, a 100 % uncertainty on the cross section is assumed. The uncertainties on these processes are large to account for kinematic effects, even though the inclusive cross sections are known to better precision.

## Results and interpretation

**Fig. 2:**
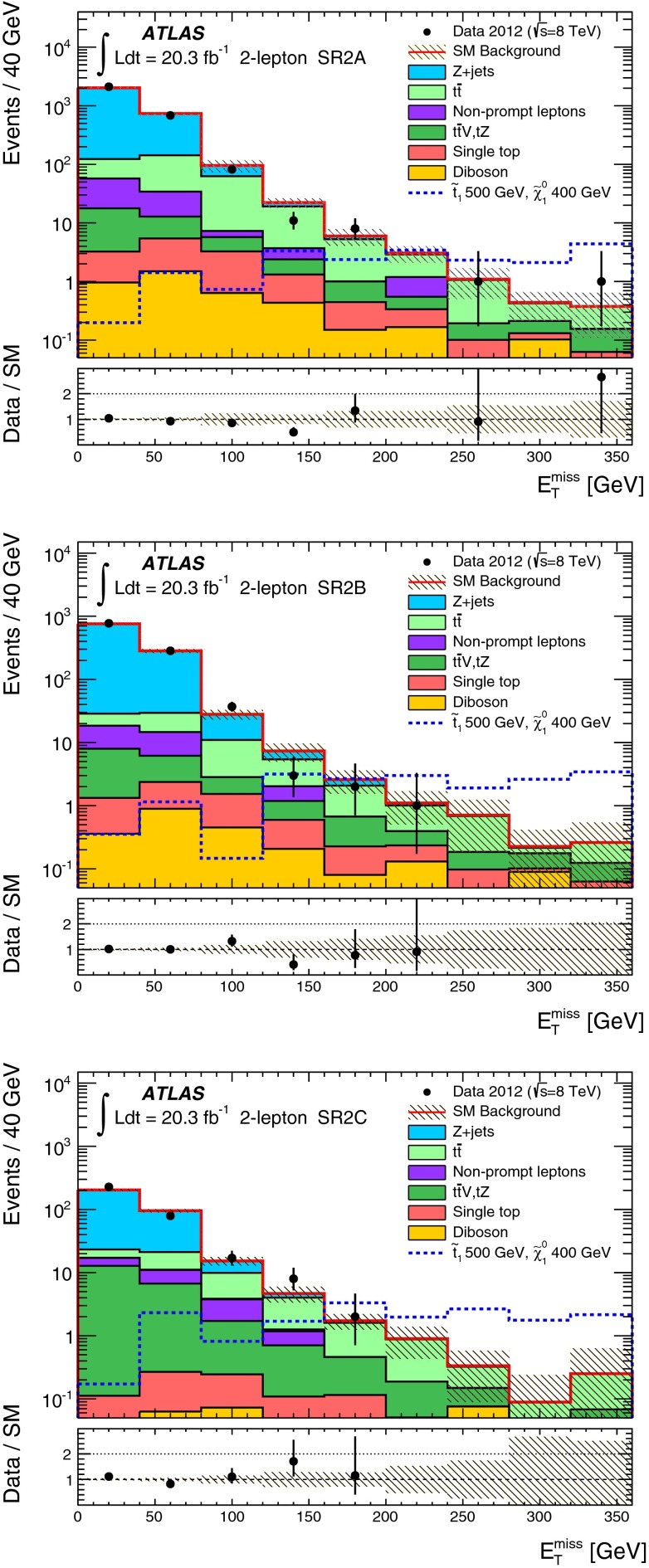
The missing transverse momentum distribution for the 2-lepton SRs SR2A (*top*), SR2B (*middle*) and SR2C (*bottom*) before the final $E_\mathrm{T}^\mathrm{miss}$ selection after the background fit. $Z$+jets distributions are obtained using the jet smearing method. *Shaded bands* denote the statistical and systematic uncertainty on the background. For illustration, distributions for a GMSB signal scenario with $m_{\tilde{t}_{1}}=500{\hbox {\ GeV}}$, $m_{\tilde{\chi }_{1}^{0}}=400{\hbox {\ GeV}}$ are shown. The last bin includes the histogram overflow

**Fig. 3:**
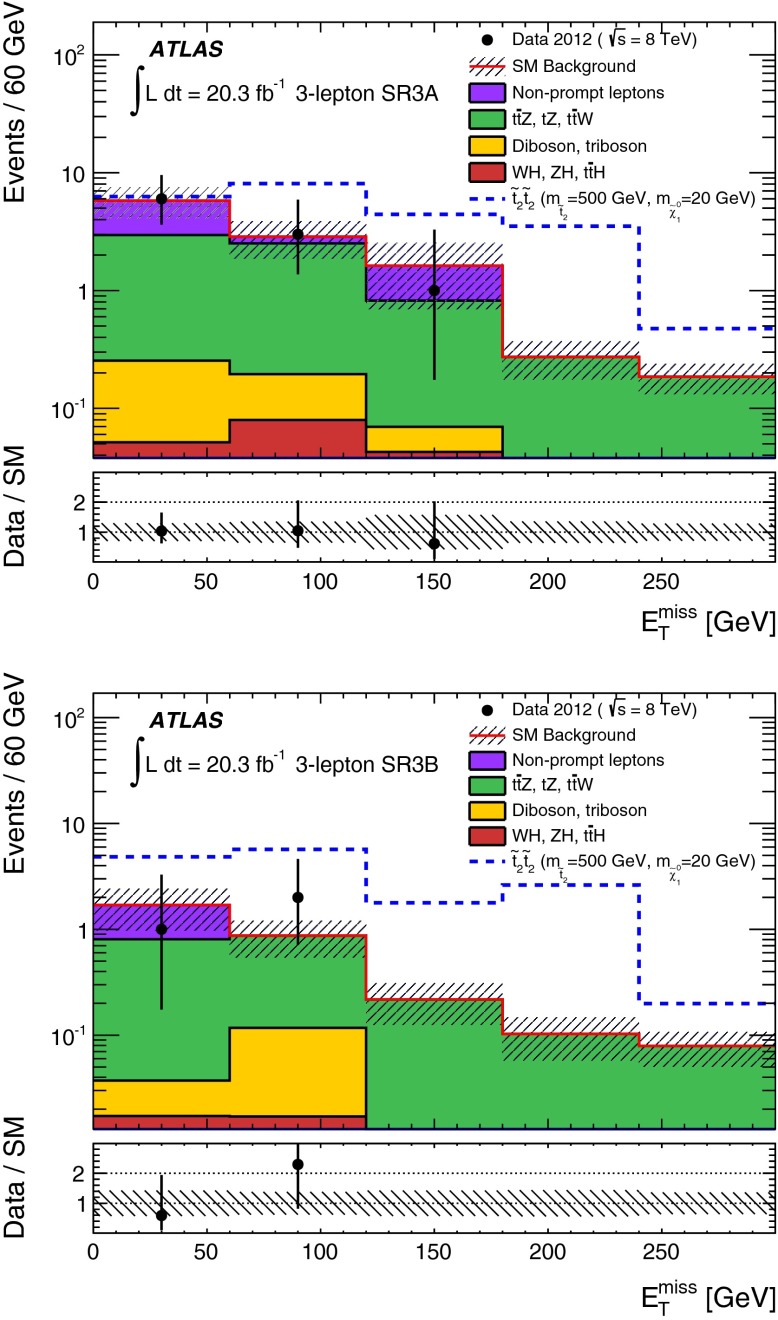
The missing transverse momentum for the 3-lepton SRs SR3A (*top*) and SR3B (*bottom*) before the final $E_\mathrm{T}^\mathrm{miss}$ selection. *Shaded bands* denote the statistical and systematic uncertainty on the background. For illustration, distributions for a signal point in the $\tilde{t}^{}_{2}$ simplified model with $m_{\tilde{t}^{}_{2}}=500$ GeV and $m_{\tilde{\chi }^{0}_{1}}=20$ GeV are also shown. The last bin includes the histogram overflow

The number of data events observed in each SR for the two-lepton and three-lepton analyses is reported in Table 4 together with the expected SM background contributions. Figs. 2 and 3 show the $E_\mathrm{T}^\mathrm{miss}$ distributions for data and background expectations for each SR.

**Table 4:** Observed event counts and predicted numbers of events for each SM background process in the SRs used in the analysis. For two-lepton SRs, background fit results and nominal MC expectations are given for comparison. The “non-prompt” category includes $t\bar{t}$, single top and $Z$+jets processes for the three-lepton SRs SR3A and SR3B. The $p$-value of the observed events for the background only hypothesis ($p_{0}$) is also shown. The value of $p_0$ is capped at 0.5 if the number of observed events is below the number of expected events

	SR2A	SR2B	SR2C
Data	10	1	2
Fitted total SM	$10.8 \pm 1.7$	$2.4\pm 0.9$	$3.5 \pm 0.5$
$p_0$	0.50	0.50	0.50
Fitted $t\bar{t}$	$7.3 \pm 1.4$	$1.4 \pm 0.7$	$2.4\pm 0.4$
Fitted single top	$0.61\pm 0.15$	$0.23\pm 0.17$	$0.10^{+0.13}_{-0.10}$
Fitted $Z$+jets	$0.91\pm 0.22$	$0.14 \pm 0.06$	$0.16 \pm 0.06$
Fitted diboson	$0.46 \pm 0.34$	$0.27\pm 0.21$	$0.15\pm 0.12$
Fitted $t\bar{t}V$, $tZ$	$1.0 \pm 0.4$	$0.38 \pm 0.18$	$0.65 \pm 0.23$
Fitted non-prompt	$0.52 \pm 0.11$	$<$0.05	$<$0.01
MC exp. total SM	$11.6$	3.0	4.8
MC exp. $t\bar{t}$	8.1	2.0	3.7
MC exp. single top	0.61	0.24	0.14
Data-driven $Z$+jets	0.88	0.13	0.18
MC exp. diboson	0.48	0.28	0.15
MC exp. $t\bar{t}V$, $tZ$	1.0	0.38	0.66
Data-driven non-prompt	0.52	$<$0.05	$<$0.01

	SR3A	SR3B	
Data	4	2	
Total SM	4.5 $\pm$ 1.4	1.3 $\pm$ 0.4	
$p_0$	0.50	0.30	
MC exp. $t\bar{t}V$, $tZ$	3.5 $\pm$ 1.2	1.1 $\pm$ 0.4	
MC exp. diboson, triboson	0.1 $\pm$ 0.1	0.1 $\pm$ 0.1	
MC exp. $Wh$, $Zh$, $t\bar{t}h$	0.1 $\pm$ 0.1	0.04 $\pm$ 0.04	
Data-driven non-prompt	0.8 $\pm$ 0.7	$<$0.2	

No excess is observed in any of the SRs. The probability ($p_0$-value) of the SM background to fluctuate to the observed number of events or higher in each SR is also reported in Table 4, and has been truncated at 0.5. Upper limits at 95 % CL on the number of beyond the SM (BSM) events for each SR are derived using the CL$_s$ prescription [92] and neglecting any possible signal contamination in the control regions. After normalising these by the integrated luminosity of the data sample, they can be interpreted as upper limits on the visible BSM cross section, $\sigma _\mathrm{vis}$, defined as the product of acceptance, reconstruction efficiency and production cross section. The limits are calculated from pseudo-experiments as well as with asymptotic formulae [86] for comparison. The results are given in Table 5.

**Table 5:** Signal model independent upper limits on the visible signal cross section ($\sigma _\mathrm{vis}=\sigma _\mathrm{prod}\times A \times \epsilon$) in the five SRs. The numbers (in parenthesis) give the observed (expected) 95 % CL upper limits. Calculations are performed with pseudo-experiments. The $\pm$1$\sigma$ variations on the expected limit due to the statistical and background systematic uncertainties are also shown. The equivalent limits on the visible cross section calculated using an asymptotic method are given inside the square brackets

Signal region	$\sigma _\mathrm{vis}$ [fb]	
SR2A	0.40 ($0.46^{+0.16}_{-0.13}$)	[0.39 ($0.41^{+0.20}_{-0.12}$)]
SR2B	0.19 ($0.24^{+0.07}_{-0.05}$)	[0.19 ($0.22^{+0.13}_{-0.05}$)]
SR2C	0.20 ($0.27^{+0.11}_{-0.07}$)	[0.20 ($0.27^{+0.13}_{-0.08}$)]
SR3A	0.30 ($0.31^{+0.14}_{-0.05}$)	[0.29 ($0.31^{+0.16}_{-0.10}$)]
SR3B	0.26 ($0.20^{+0.08}_{-0.02}$)	[0.24 ($0.20^{+0.11}_{-0.05}$)]

These results are also interpreted in the context of the models described in Sect. 1. Exclusion limits are calculated by combining the results from several exclusive SRs. For the GMSB scenarios, SR2C and SR3A are combined with the region with best expected sensitivity between SR2A or SR2B. For the $\tilde{t}^{}_{2}$ simplified models, SR2C is combined with the region with best expected sensitivity between SR3A or SR3B. For model-dependent interpretations, the fit described in Sect. 5 is modified to include the expected signal contamination of the CRs and the observed number of events in the SRs as well as an extra free parameter for a possible BSM signal strength which is constrained to be non-negative. The expected and observed exclusion limits are calculated using asymptotic formulae for each SUSY model point, taking into account the theoretical and experimental uncertainties on the SM background and the experimental uncertainties on the signal. The impact of the uncertainties on the signal cross section is also addressed for the observed limit only by showing the results obtained when moving the nominal cross section up or down by the $\pm 1\sigma$ theoretical uncertainty. Quoted numerical limits on the particle masses refer to the signal cross sections reduced by 1$\sigma$.

**Fig. 4:**
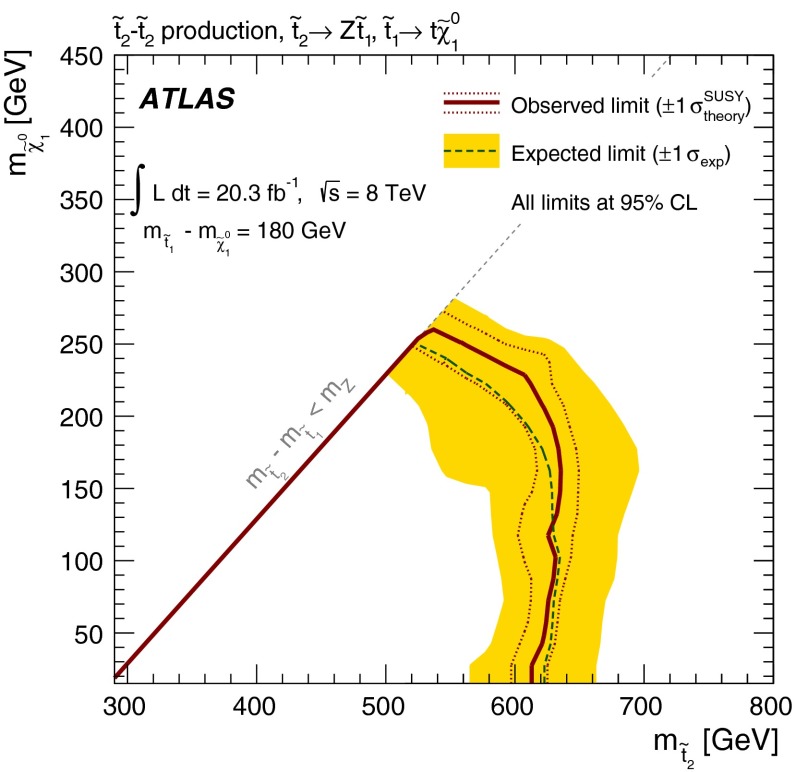
Expected and observed exclusion limits in the $m_{\tilde{t}^{}_{2}}$-$m_{\tilde{\chi }_1^0}$ plane for the direct $\tilde{t}^{}_{2}$ pair production simplified model with $\text {BR}(\tilde{t}^{}_{2}\rightarrow Z\tilde{t}^{}_{1})=1$. The contours of the band around the expected limit are the $\pm$1$\sigma$ results, including all uncertainties except theoretical uncertainties on the signal cross section. The *dotted lines* around the observed limit illustrate the change in the observed limit as the nominal signal cross section is scaled up and down by the theoretical uncertainty. All limits are computed at 95 % CL

**Fig. 5:**
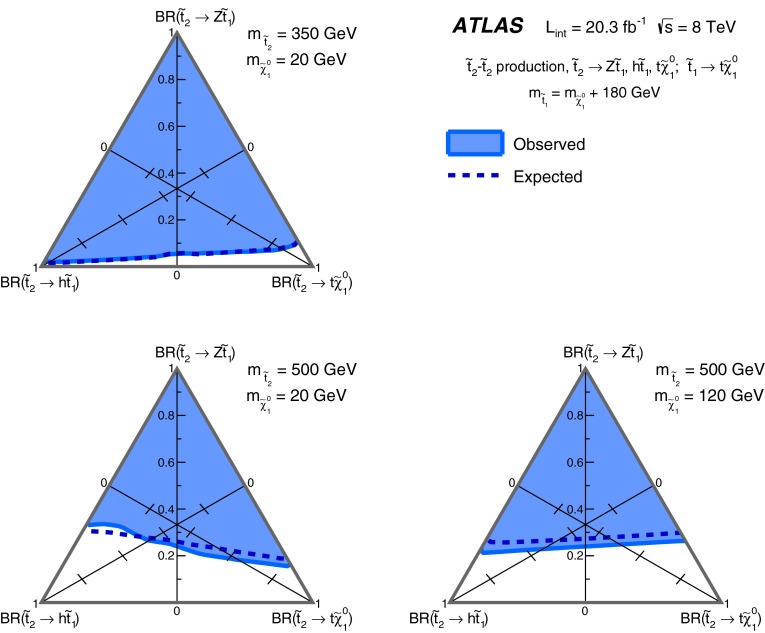
Exclusion limits at 95 % CL are shown for the direct $\tilde{t}^{}_{2}$ pair production simplified model as a function of the branching ratios $\text {BR}(\tilde{t}^{}_{2}\rightarrow Z\tilde{t}^{}_{1})$, $\text {BR}(\tilde{t}^{}_{2}\rightarrow h\tilde{t}^{}_{1})$ and $\text {BR}(\tilde{t}^{}_{2}\rightarrow t\tilde{\chi }_1^0$) for $(m_{\tilde{t}^{}_{2}},m_{\tilde{\chi }_1^0})=(350,20) {\hbox {\ GeV}}$ (*top*), $(500,20) {\hbox {\ GeV}}$ (*bottom left*) and $(500,120){\hbox {\ GeV}}$ (*bottom right*). The *dashed* and *solid lines* show the expected and observed limits, respectively, including all uncertainties except the theoretical signal cross section uncertainty (PDF and scale)

Figure 4 shows the limit obtained in the $\tilde{t}^{}_{2}$ simplified model, which excludes $m_{\tilde{t}^{}_{2}}<525{\hbox {\ GeV}}$ for $m_{\tilde{\chi }_1^0}<240{\hbox {\ GeV}}$ and $m_{\tilde{t}^{}_{2}}<600{\hbox {\ GeV}}$ for $m_{\tilde{\chi }_1^0}<200{\hbox {\ GeV}}$. The interpolation of the limit contours between the simulated points towards the $\tilde{t}^{}_{2}\rightarrow Z\tilde{t}^{}_{1}$ kinematic boundary has been established using MC generator level information. A reduction in acceptance of up to 20 % is observed in the region where $m_{\tilde{t}^{}_{2}}-m_{\tilde{t}^{}_{1}}-m_{Z}$ is comparable to the $Z$ boson width. The region with $m_{\tilde{t}^{}_{2}}-m_{\tilde{t}^{}_{1}}<m_{Z}$, where the $\tilde{t}^{}_{2}\rightarrow Z^{(*)}\tilde{t}^{}_{1}$ decay involves an off-shell $Z$, has not been considered since in that case other $\tilde{t}^{}_{2}$ decay modes, such as $\tilde{t}^{}_{2}\rightarrow t\tilde{\chi }_{1}^{0}$, would be dominant. If the assumption on the 100 % branching ratio for the $\tilde{t}^{}_{2}\rightarrow Z \tilde{t}^{}_{1}$ decay mode is relaxed, the $\tilde{t}^{}_{2}$ can also decay via $\tilde{t}^{}_{2}\rightarrow h\tilde{t}^{}_{1}$ and $\tilde{t}^{}_{2}\rightarrow t\tilde{\chi }_{1}^{0}$. Exclusion limits as a function of the $\tilde{t}^{}_{2}$ branching ratios are shown in Fig. 5 for representative values of the masses of $\tilde{t}^{}_{2}$ and $\tilde{\chi }_1^0$. For low $\tilde{t}^{}_{2}$ mass ($m_{\tilde{t}^{}_{2}}=350{\hbox {\ GeV}}$), SUSY models with $\text {BR}(\tilde{t}^{}_{2}\rightarrow Z\tilde{t}^{}_{1})$ above 10 % are excluded. For higher stop mass ($m_{\tilde{t}^{}_{2}}=500{\hbox {\ GeV}}$), models with $\text {BR}(\tilde{t}^{}_{2}\rightarrow Z\tilde{t}^{}_{1})$ above 15–30 % are excluded, with a small dependence on the value of the neutralino mass, $\text {BR}(\tilde{t}^{}_{2}\rightarrow h\tilde{t}^{}_{1})$ and $\text {BR}(\tilde{t}^{}_{2}\rightarrow t\tilde{\chi }_{1}^{0})$.

**Fig. 6:**
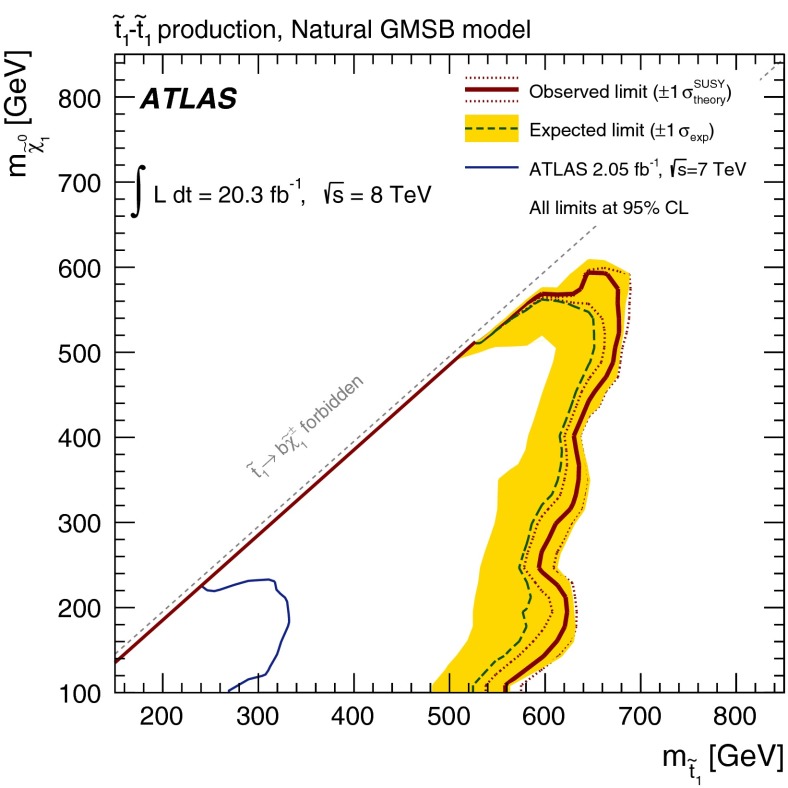
Expected and observed exclusion limits at 95 % CL for the stop natural GMSB model described in the text. The contours of the band around the expected limit are the $\pm$1$\sigma$ results, including all uncertainties except theoretical uncertainties on the signal cross section. The *dotted lines* around the observed limit illustrate the change in the observed limit as the nominal signal cross section is scaled up and down by the theoretical uncertainty. For comparison, the observed exclusion limit with 2.05 fb$^{-1}$ of data at $\sqrt{s}=7{\hbox {\ TeV}}$ at ATLAS for a similar model [34] is shown

In Fig. 6 the expected and observed limits are shown for the GMSB scenarios on the $\tilde{t}^{}_{1}$, $\tilde{\chi }_1^0$ mass plane. Stop masses up to $540{\hbox {\ GeV}}$ are excluded for neutralino masses of $100 {\hbox {\ GeV}}<m_{\tilde{\chi }_{1}^{0}}<m_{\tilde{t}_{1}}-10{\hbox {\ GeV}}$. In the parameter space region where the $\tilde{t}^{}_{1}$ only decays via $b\tilde{\chi }_{1}^{\pm }$, the exclusion extends up to stop masses of $660{\hbox {\ GeV}}$ for neutralinos of $550{\hbox {\ GeV}}$. For illustration, the exclusion limits obtained with 2.05 fb$^{-1}$ of ATLAS data at $\sqrt{s}=7{\hbox {\ TeV}}$ for the similar model are also shown, in which the maximum limit on the stop masses was $330{\hbox {\ GeV}}$. Due to the increase in statistics and the proton–proton collision energy, as well as the optimised selections for these conditions, much stronger constraints are now set on this model.

## Summary and Conclusions

This paper presents a dedicated search for direct stop pair production in decays with an experimental signature compatible with the production of a $Z$ boson, *b*-jets and missing transverse momentum. The analysis is performed with $pp$ collision data at $\sqrt{s}=8{\hbox {\ TeV}}$ collected with the ATLAS detector at the LHC corresponding to an integrated luminosity of 20.3 fb$^{-1}$. The results are interpreted in the framework of simplified models with production of $\tilde{t}^{}_{2}$ as well as in a natural GMSB model.

In a simplified model characterised by the decay chain $\tilde{t}^{}_{2}\rightarrow Z\tilde{t}^{}_{1}$ with $\tilde{t}^{}_{1}\rightarrow t\tilde{\chi }_{1}^{0}$ and the mass difference between $\tilde{t}^{}_{1}$ and $\tilde{\chi }_{1}^{0}$ slightly larger than the top mass, parameter space regions with $m_{\tilde{t}^{}_{2}}<600{\hbox {\ GeV}}$ and $m_{\tilde{\chi }_1^0}<200 {\hbox {\ GeV}}$ are excluded at 95 % CL. When the $\tilde{t}^{}_{2}\rightarrow h\tilde{t}^{}_{1}$ and $\tilde{t}^{}_{2}\rightarrow t\tilde{\chi }_1^0$ decays are included in the model, $\text {BR}(\tilde{t}^{}_{2}\rightarrow Z\tilde{t}^{}_{1})>$ 10–30 % are excluded for several mass configurations. These are the first experimental results on the search for $\tilde{t}^{}_{2}$.

In the GMSB scenario, where the $\tilde{t}^{}_{1}$ might decay to $b\tilde{\chi }_{1}^{\pm }$ or $t\tilde{\chi }_{1}^{0}(\tilde{\chi }_{2}^{0})$ and the $\tilde{\chi }_{1}^{0}$ decay in $Z\tilde{G}$ or $h\tilde{G}$, parameter space regions with $\tilde{t}^{}_{1}$ masses below $540{\hbox {\ GeV}}$ are excluded at 95 % CL for $100{\hbox {\ GeV}}< m_{\tilde{\chi }_{1}^{0}}<m_{\tilde{t}_{1}}-10{\hbox {\ GeV}}$. These limits are much stronger than those set on the similar model considered in the search at $\sqrt{s}=7{\hbox {\ TeV}}$. For $\tilde{\chi }_{1}^{0}$ masses of about $550{\hbox {\ GeV}}$, better sensitivity is achieved and $\tilde{t}^{}_{1}$ masses below $660{\hbox {\ GeV}}$ are excluded.

## Footnotes

1 ATLAS uses a right-handed coordinate system with its origin at the nominal *pp* interaction point (IP) in the center of the detector and the $z$-axis along the beam. The $x$-axis points from the IP to the center of the LHC ring, and the $y$-axis points upward.   Cylindrical coordinates $(r,\phi )$ are used in the transverse plane, $\phi$ being the azimuthal angle around the $z$-axis.   The pseudorapidity is defined in terms of the polar angle $\theta$ as $\eta =-\ln \tan (\theta /2)$. The separation between final state particles is defined as $\Delta R=\sqrt{(\Delta \eta )^2+(\Delta \phi )^2}$. The transverse momentum is denoted as $p_{\mathrm{T}}$.
